# Progressive trajectories of schizophrenia across symptoms, genes, and the brain

**DOI:** 10.1186/s12916-023-02935-2

**Published:** 2023-07-03

**Authors:** Sisi Jiang, Huan Huang, Jingyu Zhou, Hechun Li, Mingjun Duan, Dezhong Yao, Cheng Luo

**Affiliations:** 1 The Clinical Hospital of Chengdu Brain Science Institute, MOE Key Lab for Neuroinformation, School of Life Science and Technology, University of Electronic Science and Technology of China, Chengdu, 611731 People’s Republic of China; 2 Research Unit of NeuroInformation, Chinese Academy of Medical Sciences, 2019RU035 Chengdu, People’s Republic of China; 3 High-Field Magnetic Resonance Brain Imaging Key Laboratory of Sichuan Province, Center for Information in Medicine, University of Electronic Science and Technology of China, No. 2006, Xiyuan Ave., West Hi-Tech Zone, 611731 Chengdu, Sichuan People’s Republic of China

**Keywords:** Schizophrenia, Neuroimaging, Symptom, Genetics, Progression

## Abstract

**Background:**

Schizophrenia is characterized by complex psychiatric symptoms and unclear pathological mechanisms. Most previous studies have focused on the morphological changes that occur over the development of the disease; however, the corresponding functional trajectories remain unclear. In the present study, we aimed to explore the progressive trajectories of patterns of dysfunction after diagnosis.

**Methods:**

Eighty-six patients with schizophrenia and 120 healthy controls were recruited as the discovery dataset. Based on multiple functional indicators of resting-state brain functional magnetic resonance imaging, we conducted a duration-sliding dynamic analysis framework to investigate trajectories in association with disease progression. Neuroimaging findings were associated with clinical symptoms and gene expression data from the Allen Human Brain Atlas database. A replication cohort of patients with schizophrenia from the University of California, Los Angeles, was used as the replication dataset for the validation analysis.

**Results:**

Five stage-specific phenotypes were identified. A symptom trajectory was characterized by positive-dominated, negative ascendant, negative-dominated, positive ascendant, and negative surpassed stages. Dysfunctional trajectories from primary and subcortical regions to higher-order cortices were recognized; these are associated with abnormal external sensory gating and a disrupted internal excitation–inhibition equilibrium. From stage 1 to stage 5, the importance of neuroimaging features associated with behaviors gradually shifted from primary to higher-order cortices and subcortical regions. Genetic enrichment analysis identified that neurodevelopmental and neurodegenerative factors may be relevant as schizophrenia progresses and highlighted multiple synaptic systems.

**Conclusions:**

Our convergent results indicate that progressive symptoms and functional neuroimaging phenotypes are associated with genetic factors in schizophrenia. Furthermore, the identification of functional trajectories complements previous findings of structural abnormalities and provides potential targets for drug and non-drug interventions in different stages of schizophrenia.

**Supplementary Information:**

The online version contains supplementary material available at 10.1186/s12916-023-02935-2.

## Background

Schizophrenia is a severe psychiatric disorder that is characterized by positive, negative, and cognitive symptoms, which involve distributed regions in the brain [1]. A long-standing neurodevelopmental hypothesis has greatly contributed to our understanding of the development of schizophrenia [2]. The dopamine hypothesis suggests that a hyper-response occurs in schizophrenia; this is consistent with the mechanism of current antipsychotic drugs, which block dopamine D2 receptors [3, 4]. Furthermore, although no histopathological evidence has yet met the definition of neurodegeneration in schizophrenia, a neurodegenerative hypothesis has been proposed to interpret the progressive course that is observed in this disease [5]. To date, many genes have been recognized as important in different periods of the clinical course of schizophrenia [6]. People tend to seek clinical help when their symptoms meet the clinical diagnosis threshold; they usually show predominantly positive symptoms at this stage and respond well to antipsychotic drugs. Unfortunately, however, antipsychotic drugs cannot effectively control negative and cognitive symptoms, which are the main symptom types as the disease progresses [7]. Heterogeneous symptoms exist in different stages of schizophrenia, and the disease progression involves different structural and functional abnormalities.

Magnetic resonance imaging (MRI) studies have provided multimodal evidence to indicate abnormalities in distributed brain regions in schizophrenia and have highlighted the network properties of the disease [8, 9]. Many studies have investigated the detailed anatomical features of schizophrenia and provide compelling evidence of striatum-dominated atrophy and associated morphological abnormalities [10]. Furthermore, longitudinal studies of individuals with first-episode schizophrenia have demonstrated progressive gray matter loss in this disease [11]. Similarly, one of our previous studies demonstrated a progressive reduction in gray matter in patients with schizophrenia [12]. The duration of the disease and antipsychotic treatments are also associated with progressive morphological changes in the brain [13]. In resting-state functional MRI, multiple functional indicators (FIs) have been proposed to illustrate multiple-view abnormalities of brain functional activity in schizophrenia [14–16], thus providing evidence of the underlying pathology of this disease. Although abnormalities are not completely consistent across indicators, these different functional features can be referred to as representations of complex pathological mechanisms in different functional dimensions. In contrast, progressive functional changes are relatively less studied in schizophrenia. There are several possible reasons for this discrepancy: (1) the difficulties obtaining long-term longitudinal functional data from large samples, (2) the existence of large functional heterogeneity in patients with schizophrenia, and (3) the relatively poor stability of functional signals relative to structural data.

It would be interesting to explore the intrinsic characteristics of the disease itself through multiple FIs. A prior meta-analysis of multiple FIs integrated findings across publications in an attempt to identify duration-associated functional features [17]. However, correlation analyses in a cross-sectional analysis are unable to reveal specific patterns of dysfunction over different disease courses. Additionally, although a longitudinal analysis is the best way to reveal the progression of a disease, decades of longitudinal data are extremely difficult to obtain. There is thus a need to develop an alternative research framework to address this issue. Moreover, to establish macro- and micro-scale understanding of diseases, studies have linked neuroimaging profiles with gene transcriptomic data across psychiatric disorders [18, 19]. Gray and white matter microstructures are associated with polygenic risk for schizophrenia and have been further suggested to affect the psychiatric symptoms of patients [20, 21]. Moreover, associations between genotypes and clinical phenotypes are complex over different periods of schizophrenia [2, 22]. Therefore, integrating neuroimaging and gene transcriptomic data may provide further insights into the pathological mechanisms of the disease.

To address these issues, we used a longitudinal-substituted approach to investigate the progressive dysfunction that occurs in schizophrenia using cross-sectional datasets. This approach was conducted using a duration-sliding dynamic analysis framework, in which multiple FIs were integrated to characterize whole-brain voxel-wise dysfunction. Affinity propagation clustering and Liptak–Stouffer approaches were then used to acquire duration-labeled specific stages of dysfunction in the disease. We hypothesized that gene expression levels would be related to the patterns of dysfunction as well as disease progression. We therefore used a regression model to identify dysfunction-associated genetic factors in distinct stages using the Allen Human Brain Atlas (AHBA) database [23]. Moreover, enriched networks were identified based on merged genes across stages to uncover schizophrenia-related pathways using Metascape [24]. The overall aim of the present study was to identify disease-related multi-trajectories, from symptoms to neuroimaging to genes. The study flow chart is illustrated in Additional file 1: Fig. S1.

## Methods

### Participants

For this study, 86 patients with schizophrenia were recruited from the Clinical Hospital of Chengdu Brain Science Institute. Each patient was diagnosed based on the Diagnostic and Statistical Manual of Mental Disorders, fourth edition. Subjects with a history of brain injuries, substance-related disorders, or major medical or neurological disorders were excluded. Symptoms were evaluated using the Positive And Negative Syndrome Scale (PANSS). A standardized quantitative formula was used to evaluate the chlorpromazine equivalents for each antipsychotic medication [25]. As controls, 120 healthy individuals without neurological or psychiatric disorders were recruited. This study was approved by the research ethics committee of Chengdu Mental Health Center with the approval number CDMHLL-2017008, and written informed consent was obtained from participants (or their legal guardians if they were under 18 years old) in accordance with the Declaration of Helsinki. Detailed demographic and clinical information is shown in Table 1.

**Table 1:** Study cohort demographics of the schizophrenia and control participants

Characteristic	Schizophrenia (*n* = 86)	HC (*n* = 120)	*P*-value
Age (years)	40.66 ± 11.17	37.73 ± 14.69	0.12
Gender (M:F)	61:25	80:40	0.52
Education	11.45 ± 2.59	11.07 ± 3.08	0.36
Duration (years)	15.22 ± 10.20	–	–
Chlorpromazine equivalents (mg/day)	337.96 ± 144.41	–	–
PANSS score			
Total	62.21 ± 13.16	–	–
Positive	13.32 ± 5.84	–	–
Negative	20.70 ± 6.01	–	–
General	28.19 ± 5.81	–	–

*F* female, *HC* healthy control, *M* male, *PANSS* Positive And Negative Syndrome Scale

### Data acquisition

MRI images were acquired on a 3-T scanner equipped with an eight-channel phased-array head coil (EXCITE, GE Healthcare, Milwaukee, WI, USA). An echo-planar imaging sequence was used for resting-state functional data (echo time [TE] = 30 ms, repetition time [TR] = 2000 ms, data matrix = 64 × 64, field of view = 24 cm × 24 cm, flip angle [FA] = 90°, slice thickness = 4 ms [no gap], and 32 axial slices in each volume). All subjects were asked to close their eyes without falling asleep during the scan. Each scan lasted 400 s and generated 200 volumes. A three-dimensional fast spoiled gradient-echo sequence was used to acquire axial anatomical T1-weighted images, with the following parameters: TE = 3.2 ms, TR = 8.2 ms, field of view = 25.6 cm × 25.6 cm, data matrix = 256 × 256, flip angle = 12°, and thickness = 1 mm (no gap).

### Dysfunction in association with disease progression

The preprocessing of functional MRI and structural data followed the procedures detailed in our previously published articles (Additional file 1: Method S1 [26]). Five FIs were used to describe the overall characteristics of brain function: the fractional amplitude of low-frequency fluctuation, regional homogeneity, and functional connectivity density at global, local, and long-range levels. Progressive dysfunction was assessed using the following analysis. Patients with schizophrenia were grouped according to disease duration. Next, an approach similar to an overlapping sliding window in the dynamic analysis was used to divide subgroups [27, 28].

Specifically, we divided patients into different groups with overlap according to their duration of disease. Taking 5 years as the window length, patients with a course of 1 to 5 years were classified into group 1, patients with a course of 2 to 6 years were classified into group 2, patients with a course of 3 to 7 years were classified into group 3, and so on. In each subgroup, whole-brain voxel-wise FIs were calculated and compared with those of healthy controls using a two-sample *t*-test, with age, sex, total intracranial volume, and head motion included as nuisance covariates (Additional file 1: Method S2). To capture duration-specific case–control differences, an affinity propagation clustering approach was applied to create case–control *t*-maps of all subgroups for each FI [29] (Additional file 1: Method S3 [30, 31]). Each FI then uncovered a set of duration-related functional states, which characterized schizophrenia-related disturbances from a specific perspective. To identify progressive stages using multifunctional characteristics, states from distinct FIs were aligned according to maximum spatial similarity and maximum temporal overlap (Additional file 1: Method S4 and Fig. S2). After clustering and alignment across states, patterns of dysfunction in the progressive stages were identified using the Liptak–Stouffer method [32–34] (Additional file 1: Method S5 [32–34]). Moreover, to quantify the progressive patterns of dysfunction, we used cerebral functional gradient maps [35] and calculated the spatial correlations between schizophrenia stage-related cortical dysfunction and cortical hierarchy.

### Associations between dysfunction and behavior

To identify the associations between stage-specific dysfunction and PANSS scores, we conducted partial linear squares regression (PLSR) for each stage. Specifically, we parcellated the whole brain into 246 regions and extracted the FIs of each region by averaging all voxels using a previously defined atlas [36]. In this way, a 246 × 5 FI matrix was generated for each subject as the neuroimaging variables, and positive, negative, and general PANSS scores were taken as the behavioral variables. We concatenated all subjects in each stage and constructed a regression of the responses in behavioral variables on the predictors in neuroimaging variables. A non-parametric technique based on randomly shuffling subjects (5000 times) was used for the inferential analysis of PLSR results, which tests whether the explained variance of PLS1 is significantly more than that expected by chance (permutation test, *p*_perm_ < 0.001). The current study focused on the combination (PLS1) mostly associated with behavioral variables. The behavioral variables were correlated with the neuroimaging PLS1 scores to reveal the specific associations between neuroimaging and behavioral characteristics. We also compared the PANSS scores between stages using a permutation test, which randomly divides patients into two groups (5000 times) and calculates the differences to generate the null model. The significance of observed differences in PANSS scores between the stages was determined using a comparison with the permutated distribution.

We also detected the network- and region-level significance of neuroimaging PLS1 loadings. In this analysis, eight previously defined brain networks were used to illustrate network-level significance: the visual network, sensorimotor network, salience network, dorsal attention network, limbic network, frontoparietal network, default mode network, and subcortical network. We averaged the loadings of all FIs of all regions belonging to each network, thus generating eight network-level neuroimaging loadings. Additionally, we investigated the significance of the loadings (averaged across FIs) in regions with abnormal FIs. We used regions in which more than 30% of voxels had significantly abnormal FIs in schizophrenia.

### Genome expression data

The AHBA database (http://human.brain-map.org) provides gene expression data from the brains of six healthy adult human donors, with 3702 spatially resolved samples. In this database, each donor’s brain has been normalized and is divided into 246 smaller contiguous regions (atlas) with approximately homogeneous sizes. In the present study, we only analyzed the left hemisphere data from the six donors (because data from the right hemisphere are included for only two donors in the AHBA database). We thus obtained the estimated expression values for each of the 10,027 genes in 123 regions of the left hemisphere, as a 123 × 10,027 regional transcription matrix (Additional file 1: Method S6 [18, 19, 36, 37]).

### Dysfunction-associated genes

The patterns of dysfunction in the progressive and non-progressive analyses were extracted to identify dysfunction-associated genes. The PLSR model was used to explore dysfunction-associated genome expression (transcriptional activity for all 10,027 genes). In the PLSR analysis, which links neuroimaging data with transcriptomic data, the inflation of false-positive findings may be observed because of spatial auto-correlation [38]. To account for spatial auto-correlation, a spin test based on spherical rotations (5000 times) was used for the inferential analysis of the PLSR results (permutation test, *p*_spin_ < 0.001) [39]. The spin test generates surrogate brain maps that are matched to an empirical brain for spatial autocorrelation, thus controlling for spatial contiguity and hemispheric symmetry. We also applied a bootstrapping approach, which resampled using the replacement of the cortical regions to estimate the error on the PLS weights for each gene. The weight of each gene was then transformed to a *z*-score by its ratio to its bootstrap standard error. Next, genomes with a significant contribution to case–control dysfunction were selected (*p* < 0.05, false discovery rate [FDR] corrected).

The significant genes in PLS1 overlapped with 52 schizophrenia-related genes from “genes characterized by in situ hybridization in 1000 gene survey in cortex” in the AHBA database (help.brainmap.org/display/humanbrain/Documentation) (Additional file 1: Method S7 [36]). The expression of these overlapping genes was then correlated to case–control statistical maps using Pearson’s correlation (*p* < 0.05, FDR corrected).

### Enrichment analysis

The Metascape tool (http://metascape.org/) was used to conduct the Kyoto Encyclopedia of Genes and Genomes (KEGG) enrichment analysis for the PLS1 genes that were selected from the PLSR components. For the statistical *z*-map of each stage, dysfunction-associated genes were obtained according to significant PLS1 + and PLS1 − values (|*Z*|> 5, all FDR-corrected *p* < 0.05). Each stage produced one list of genes, thus generating multiple lists of genes. To identify enriched networks related to disease progression, these multiple lists of genes were merged using Metascape. In the progressive analysis, the PLS1 − and PLS1 + gene lists were generated separately for the enrichment analyses.

### Association between dopamine and dysfunction

Open positron emission tomography (PET) and single-photon emission computerized tomography (SPECT) data from unrelated healthy controls were used (available at the NITRC website: http://www.nitrc.org/projects/spmtemplates/), including [^18^F] FDOPA PET and [^123^I] FP-CIT SPECT templates. Respective dopamine synthesis capacity and dopamine transporter values were then indexed from these templates. We also used an [^11^C] raclopride PET template from a previous study [40] to index striatal D2/D3 receptors. All templates were yielded by averaging across subjects. In the present study, spatial correlations between the case–control *t*-maps and the three dopaminergic templates were calculated (*p* < 0.05). The case–control *t*-maps of FIs and progressive stages were spatially correlated to the spatial patterns of dopamine features, including dopamine synthesis, dopamine transporters, and D2 receptors.

### Non-progressive analysis

A non-progressive analysis was conducted by comparing the FIs of all patients with those of healthy controls. The dysfunction-associated genes, enriched networks, and associations between dopamine and dysfunction were all investigated in this non-progressive analysis (Additional file 1: Method S8).

### Validation analysis

We performed three additional analyses to validate the findings from the present study. First, we used a dataset from a replication cohort of patients with schizophrenia from the University of California, Los Angeles (UCLA) (from OpenNeuro: http://openneuro.org) [41]. With this dataset, we calculated the case–control differences of FIs and identified the enriched networks associated with neuroimaging profiles. Second, the number of clusters was validated using a conventional *K-*means clustering approach. Third, different window lengths were used in the affinity propagation clustering analysis to validate the reliability of progressive dysfunction. For the detailed processing methods, see Additional file 1: Method S9 [42].

### Control analysis

The main factors that affect FIs in the brain are the morphological profiles of the brain and antipsychotics. We therefore correlated disease duration with morphological features of the brain and conducted comparisons between subgroups with short and long disease durations. The included morphological features were gray matter volume, white matter volume, and cerebrospinal fluid volume. Additionally, drug equivalents were compared between stages using a permutation test. We also evaluated the correlations between FIs and antipsychotics, and compared the FIs between subgroups with high and low drug equivalents (Additional file 1: Method S10).

## Results

### Symptom trajectory across stages

**Fig. 1:**
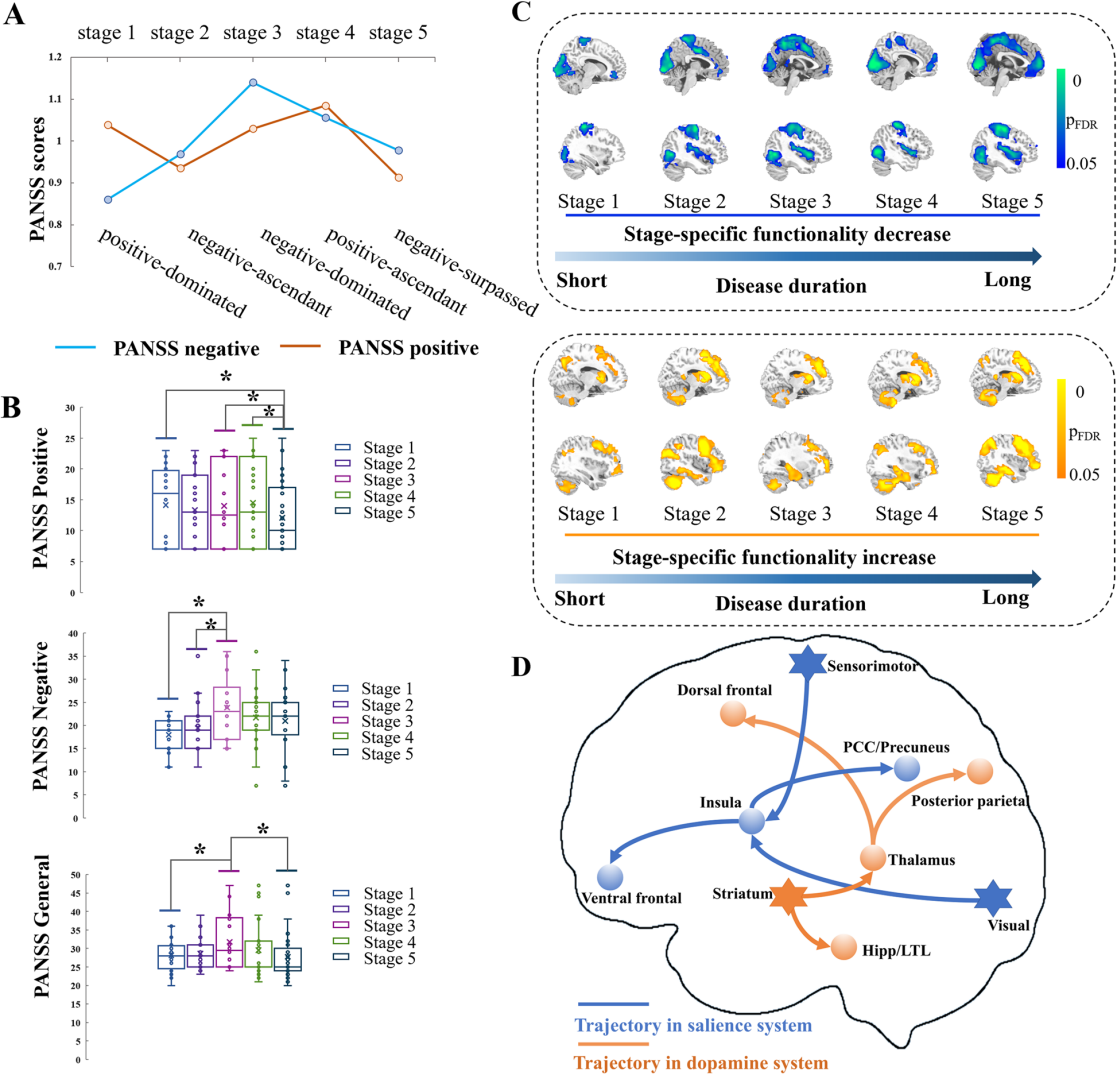
Symptoms and functional profiles in the progressive stages. **A** Using PANSS scores, we identified the positive-dominated stage 1, the negative-ascendant stage 2, the negative-dominated stage 3, the positive-ascendant stage 4, and the negative-surpassed stage 5. The PANSS general scores presented a slow uphill phase and a slow downhill phase. **B** Comparisons of PANSS scores between stages using permutation tests. *p < 0.05 (FDR corrected). **C** With disease progression, FIs progressively decreased from lower-order cortices to the insula and then to higher-order cortices in the default mode network (top middle of the panel), and FIs progressively increased from the subcortical regions to the thalamus/hippocampus and then to higher-order cortices in the frontoparietal network (bottom middle of the panel). **D** Summarized progressive dysfunction trajectory in patients with schizophrenia after diagnosis. Asterisks denote regions involved at the beginning of the disease progress and circles denote regions involved as the disease progresses. Warm colors indicate hyperfunction and cold colors indicate hypofunction

The present study generated 26 progressive subgroups based on disease duration (Additional file 2: Table S1). PANSS scores (positive, negative, general, and total) in each subgroup are shown in Additional file 2: Fig. S3. Five stages were identified, with characteristic clinical symptoms: the positive-dominated stage 1, the negative-ascendant stage 2, the negative-dominated stage 3, the positive-ascendant stage 4, and the negative-surpassed stage 5 (Fig. 1A). Differences in PANSS between stages were also observed (*p*_perm_ < 0.001) (Fig. 1B). There were significantly higher positive scores in stages 1, 3, and 4 than in stage 5. Furthermore, stage 3 had higher negative scores than stages 1 and 2 and higher general scores than stages 1 and 5.

### Dysfunction trajectories across stages

The optimal number of clusters was selected using the largest Calinski–Harabasz index score (Additional file 2: Fig. S4). The case–control *t*-maps of fractional amplitude of low-frequency fluctuation, regional homogeneity, and global and local functional connectivity densities of each subgroup were clustered into five representative disease progression states, which resulted in 4 × 5 functional states. The long-range functional connectivity density subgroup case–control *t*-maps were clustered into six representative states. Thus, we finally obtained 4 × 5 (4 features, 5 states) plus 1 × 6 (1 feature, 6 states) representative case–control differences. The conventional *k*-means clustering approach also suggested a consistent selection of cluster number (Additional file 2: Fig. S5). Representative states from different FIs were aligned, resulting in five disease progression stages. Each stage was duration-labeled: stage 1—mean 3.66 (range: 0–8) years; stage 2—mean 8.38 (range: 3–14) years; stage 3—mean 14.17 (range: 10–18) years; stage 4—mean 18.18 (range: 13–23) years; and stage 5—mean 23.32 (range: 17–30) years.

To recognize the specific pattern of dysfunction of each stage, stage-specific case–control *z*-maps were created using the Liptak–Stouffer method (*p* < 0.05, FDR corrected). Decreased FIs were persistently observed in the sensorimotor and visual cortices from stages 1 to 5. Hypofunction of the insula was observed persistently from stages 2 to 5. Notably, a cluster in the ventral medial prefrontal cortices was observed in stages 1, 2, and 3; this cluster gradually expanded in stages 4 and 5 (Fig. 1C). Increased FIs in the striatum, dorsal prefrontal cortices, and cerebellum were observed across all stages and had a gradually expanding spatial scope. Furthermore, hyperfunction of both the thalamus and hippocampus was observed at stage 2 and gradually expanded with increasing stages. Two trajectories of dysfunction were therefore summarized in schizophrenia (Fig. 1D). With window widths of 6 and 7 years (Additional file 2: Method Tables S2-S3), four stages were obtained. Although the number of stages changed, we also clearly observed a hyperfunction trajectory from subcortical areas to higher-order cortices in the frontoparietal network and a hypofunction trajectory from lower-order sensory regions to higher-order cortices in the default mode network (Additional file 2: Figs. S6-S7). Moreover, we found that the progressive dysfunction did not linearly correlate with cerebral functional gradients (Additional file 2: Fig. S8). Notably, the relative ratio of correlations with gradient 1 (demeaned) and gradient 2 (demeaned) showed the same trend as that of PANSS positive scores across the stages.

### Progressive association between dysfunction and symptoms

**Fig. 2:**
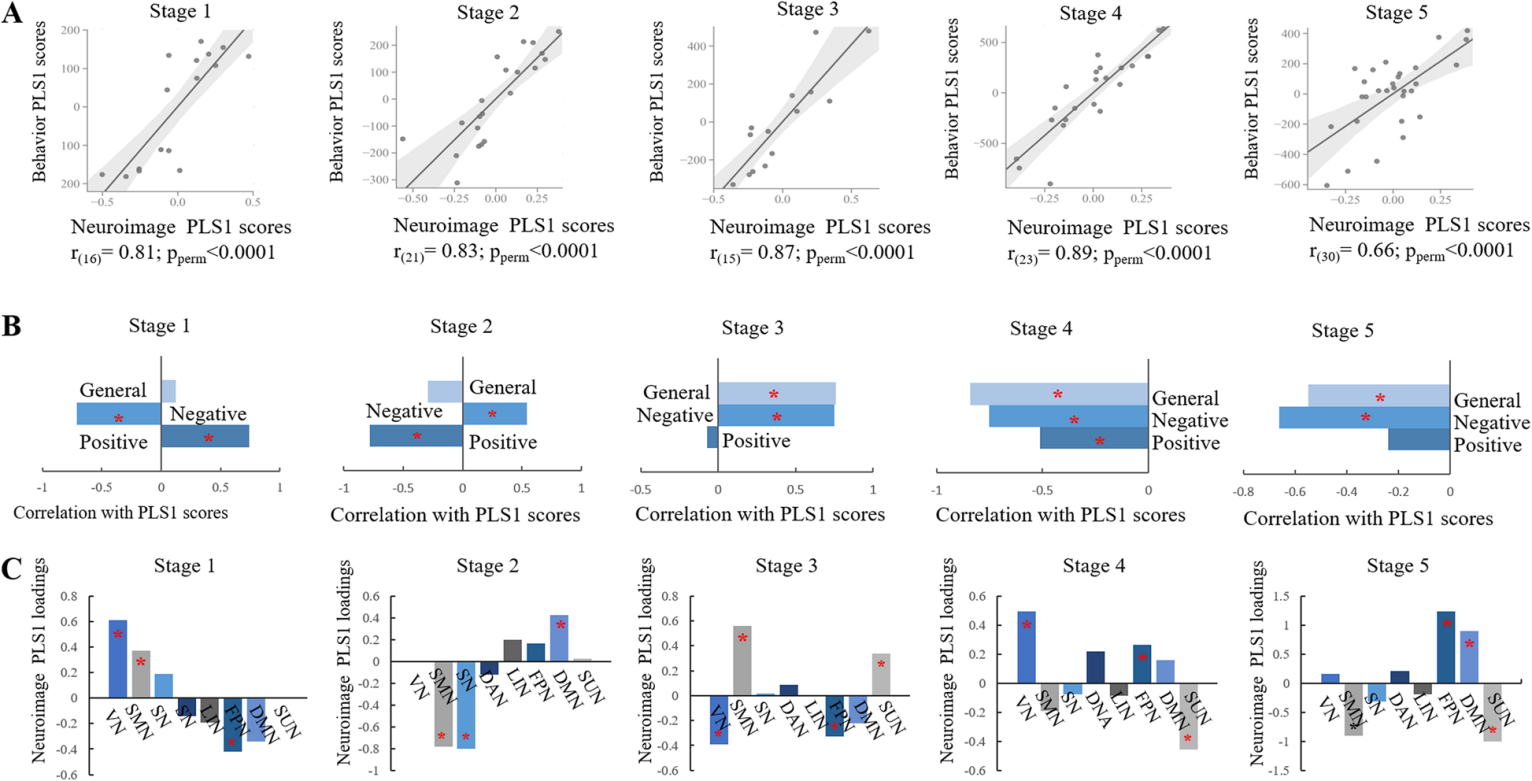
Stage-specific associations between PANSS and dysfunction. **A** Associations between neuroimaging and behavioral PLS1 scores in each stage. **B** Correlations between PANSS positive, negative, and general scores and neuroimaging PLS1 scores. **C** Network-level neuroimaging PLS1 loadings at each stage. *p < 0.05 (permutation test)

In all stages, the neuroimaging PLS1 scores were significantly positively correlated with the behavioral PLS1 scores (*p* < 0.0001) (Fig. 2A, B). In the first two stages, both the PANSS positive and negative scores significantly correlated with the neuroimaging PLS1 scores. Furthermore, in stages 3 and 5, the PANSS negative and general scores correlated with the neuroimaging PLS1 scores. The PANSS positive, negative, and general scores all significantly correlated with the neuroimaging PLS1 scores. Moreover, the neuroimaging PLS1 loadings of brain networks showed distinct association in distinct stages (*p* < 0.0001) (Fig. 2C). In the first stage, the visual and sensorimotor networks were predominantly significant, whereas the salience network had significant contributions in stage 2. In the last stage, the frontoparietal, default mode, and subcortical networks had significant loadings in the association between neuroimaging FIs and behavioral PANSS scores. The region-wise neuroimaging PLS1 loadings showed a similar trend of significance, from primary to higher-order cortices and subcortical regions, as the disease progressed (Additional file 2: Fig. S9).

In the control analyses, there were no correlations between disease duration and drug equivalent (Additional file 2: Fig. S10A). In addition, there were no differences in drug equivalent among the stages (*p* > 0.05) (Additional file 2: Fig. S10B). However, patients with high drug equivalents showed hyperfunction in the visual cortices compared with patients with low drug equivalents (Additional file 2: Figs. S11-S12). Furthermore, reduced gray matter volume and increased cerebrospinal fluid volume were observed with longer disease durations (Additional file 2: Fig. S13). The case–control *z*-maps of FIs and progressive stages were significantly positively correlated with the spatial patterns of dopamine synthesis, dopamine transporters, and D2 receptors (Additional file 2: Figs. S14-S15).

### Dysfunction-related genetic factors

**Fig. 3:**
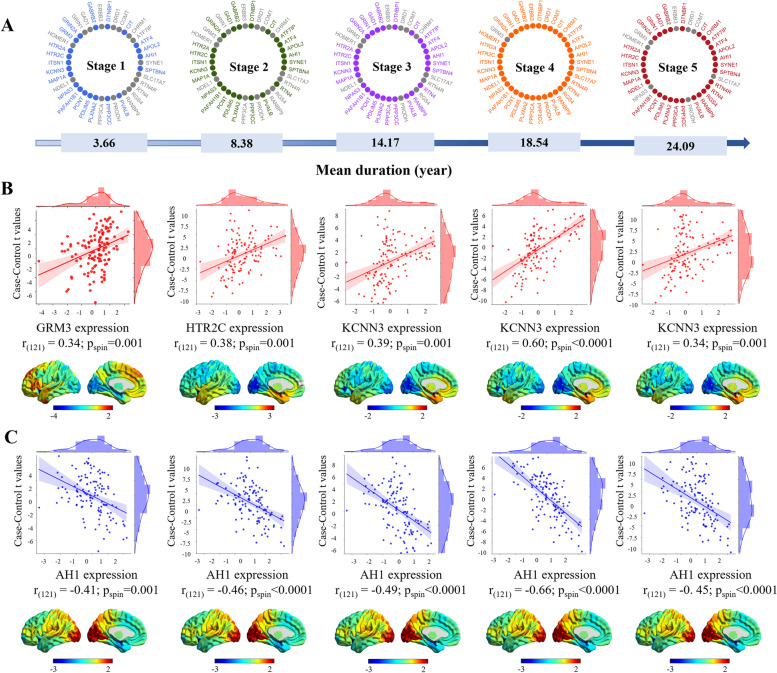
Dysfunction-associated genetic profiles in the progressive stages. **A** Overlap between the progressive dysfunction-associated genes and the schizophrenia-related genes from the AHBA in situ hybridization data. A duration range was recognized for each stage. Thirty-eight related genes were identified across all stages. Each stage is linked with distinct genes (presented in different colors). Unrelated genes are shown in gray. **B** Positive correlations (with the most significant correlations) between associated gene expression and case–control* t*-maps. **C** Negative correlations (with the most significant correlations) between associated gene expression and case–control *t*-maps

The statistical *z*-maps of the progressive stages significantly correlated with their PLS1 maps (Additional file 2: Fig. S16). In stages 1, 2, 3, 4, and 5, the PLS1 contained 21, 24, 27, 37, and 30 of the 52 schizophrenia-related genes from the AHBA in situ hybridization data, respectively. These included 15, 16, 20, 28, and 24 genes that negatively correlated with case–control *z*-maps and 6, 8, 7, 9, and 6 genes that positively correlated with case–control *z*-maps, respectively. Notably, the expression levels of *KCNN3*, *HTR2C*, and *GRM3* were the most positively correlated with the case–control *z*-maps. In contrast, the expression level of *AH1* was the most negatively correlated with the case–control *z*-maps in the five stages (*p* < 0.05) (Fig. 3).

### Enrichment networks related to dysfunction

**Fig. 4:**
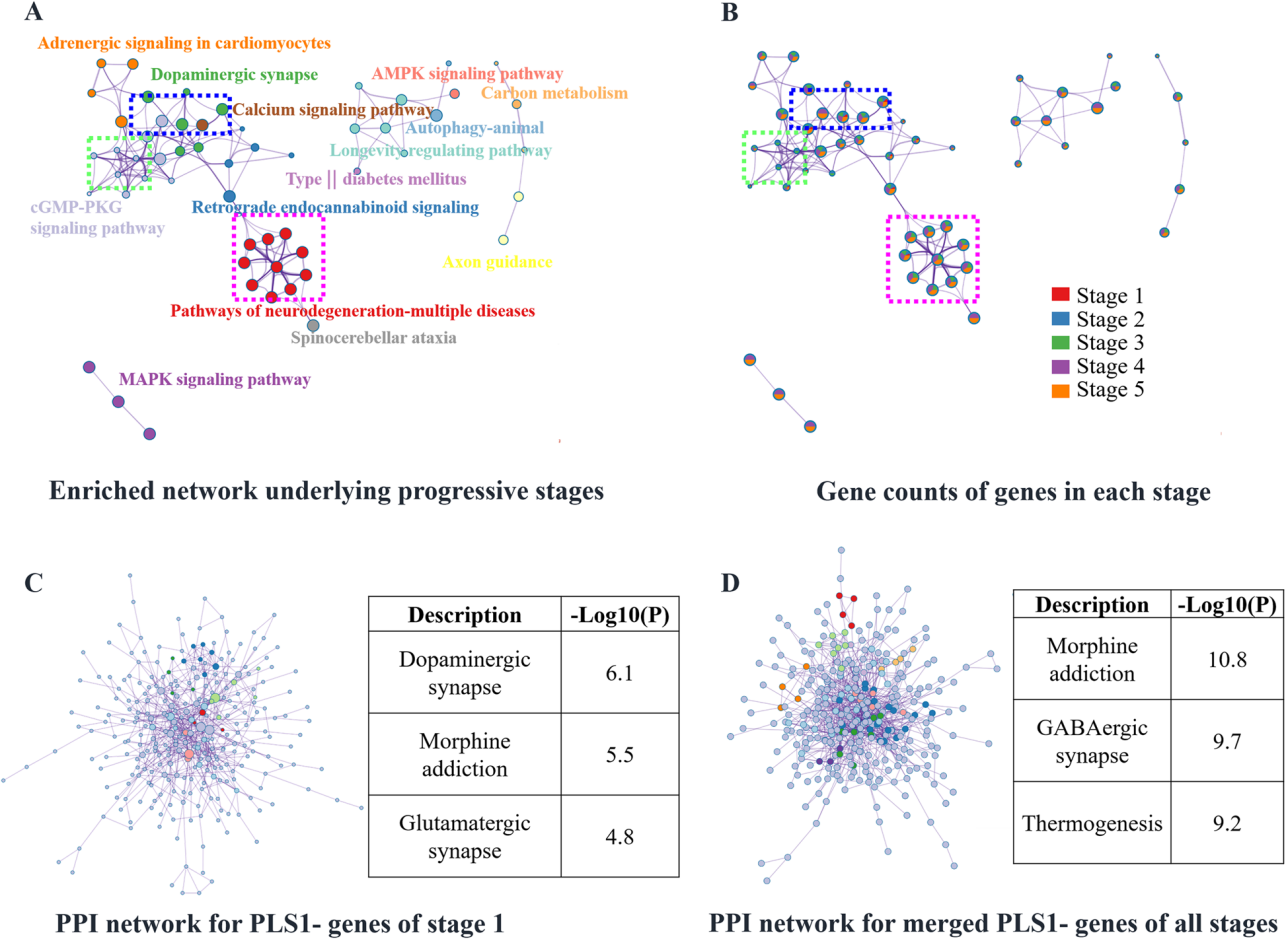
Enrichment analysis of PLS1 − genes related to disease progression. **A** Enrichment network using merged PLS1 − genes across all stages. **B** The same enrichment network as in **A** with nodes as pie charts. Each pie sector is proportional to the number of hits originating from a gene list. The color code for the pie sector represents the gene list of a certain stage. As shown in the blue dotted box, the enriched term “dopaminergic synapse” originates from the gene list of stage 1, whereas the green dotted box indicates the enriched term “cGMP-PKG signaling pathway,” which mainly originated from genes from stages 2 and 3. The pink dotted box shows that “pathways of neurodegeneration-multiple diseases” was enriched from the gene lists of stages 3, 4, and 5. **C** Protein–protein interaction network using the PLS1 − genes of stage 1, including six MCODE networks. Each color represents an MCODE network. Gene Ontology enrichment analysis was applied to each MCODE network to extract “biological meanings” from the network component, and the top three terms (with the smallest *p*-values) were retained: “dopaminergic synapse,” “morphine addiction,” and “glutamatergic synapse.” **D** Protein–protein interaction network enriched with the merged PLS1 − gene lists of all stages, including nine MCODE networks. The terms “morphine addiction,” “GABAerigic synapse,” and “thermogenesis” (the three terms with the smallest *p*-values) were extracted using Gene Ontology enrichment analysis for each MCODE. Each color represents an MCODE network

Using Metascape, stage-specific PLS1 + , and PLS1 − genes were selected to form multi-gene lists for KEGG enrichment analyses. Enriched terms across the enrichment results of PLS1 − genes are shown in Fig. 4A. For the PLS1 − genes, the enrichment network included “dopaminergic synapse,” “pathways of neurodegeneration-multiple diseases,” “calcium signaling pathway,” “cGMP-PKG signaling pathway,” and “AMPK signaling pathway.” According to the gene counts for each enriched term (Fig. 4B and Additional file 2: Fig. S17), stage 1 contributed to “dopaminergic synapse,” and stages 2–4 contributed to “pathways of neurodegeneration-multiple diseases.” In addition, neurodevelopment-related terms were observed in all stages of disease progression. A protein–protein interaction network was enriched in stage 1 (Fig. 4C) with the significant enrichment terms “dopaminergic synapse,” “morphine addiction,” and “glutamatergic synapse.” Moreover, a protein–protein interaction network was enriched for multiple PLS1 − gene lists across all stages (Fig. 4D), resulting in nine Molecular Complex Detection (MCODE) networks. The “GABAergic synapse,” “morphine addiction,” “MAPK signaling pathway,” and “PI3K-Akt signaling pathway” terms were enriched in this protein–protein interaction network. For the enrichment results of PLS1 + genes, see Additional file 2: Fig. S18.

### Non-progressive findings

Decreased FIs were identified in the sensorimotor and visual cortices, whereas increased FIs existed in the dorsolateral and medial prefrontal cortices (Additional file 3: Fig. S19). Case–control differences in FIs also revealed important associations with the expression of *AH1*, *KCNN3*, *HTR2C*, *SPTBN4*, and *PVALB* (Additional file 3: Fig. S20). “Dopaminergic synapse,” “Alzheimer’s disease,” and “pathways of neurodegeneration-multiple diseases” terms were enriched using merged PLS1 − genes, and “pathways in cancer” and “cell adhesion metabolism” terms were enriched with the highest significance using merged PLS1 + genes (Additional file 3: Fig. S21).

**Fig. 5:**
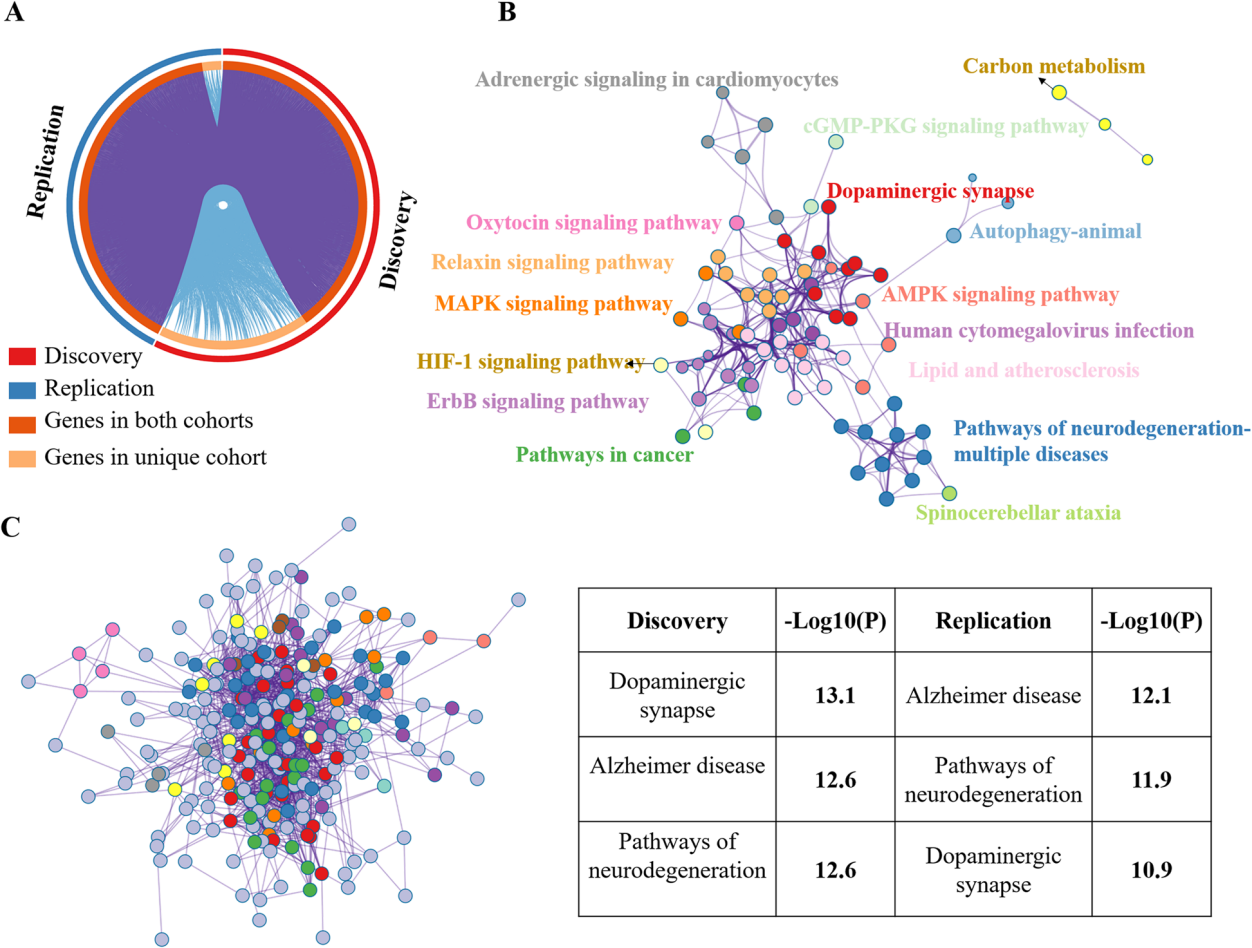
Enriched networks in the discovery and replication cohorts. **A** Overlapping genes between the discovery and replication cohorts. **B** Enrichment network using merged PLS1 (|*Z*|> 5) genes from the discovery and replication cohorts. The top three terms (with the smallest *p*-values) were “dopaminergic synapse” (− log_10_(*p*) = 12.39), “pathways of neurodegeneration-multiple diseases” (− log_10_(*p*) = 11.40), and “pathways in cancer” (− log_10_(*p*) = 10.85). **C** Protein–protein interaction network using merged PLS1 (|*Z*|> 5) genes, including 13 MCODE networks. Each color represents one MCODE network. The top right table shows the top three enriched terms, which were identified using Gene Ontology enrichment analysis for each MCODE network

Both the case–control differences in FIs and enriched networks in the replication cohort were in line with the findings from the discovery cohort (Additional file 3: Figs. S22-S23). The discovery and replication cohorts showed highly overlapping dysfunction-associated genes (Fig. 5A). Moreover, both the discovery and replication datasets were well clustered and contributed to shared enriched networks. Multi-gene lists from the discovery and replication cohorts resulted in an enriched network with consistent enrichment terms. The top three terms (with the smallest *p*-values) were “dopaminergic synapse” (− log_10_(*p*) = 12.39), “pathways of neurodegeneration-multiple diseases” (− log_10_(*p*) = 11.40), and “pathways in cancer” (− log_10_(*p*) = 10.85) (Fig. 5B). Moreover, a protein–protein interaction network was enriched, in which the top three terms (with the smallest *p*-values) were “dopaminergic synapse” (− log_10_(*p*) = 13.1 and 10.9 in the discovery and replication cohorts, respectively), “pathways of neurodegeneration-multiple diseases” (− log_10_(*p*) = 12.6 and 11.9 in the discovery and replication cohorts, respectively), and “Alzheimer’s disease” (− log_10_(*p*) = 12.6 and 12.1 in the discovery and replication cohorts, respectively) (Fig. 5C).

## Discussion

In schizophrenia, longitudinal studies of the prodromal phase to the first episode are important for revealing the pathological mechanisms of the disease, as well as a basis for its clinical treatment [43]. After being diagnosed, the vast majority of people with schizophrenia experience a lifelong regimen of alternating positive and negative symptoms or negative-dominated symptoms [1]. Subtypes of schizophrenia can be classified according to the brain functional characteristics of patients, which may lead to the application of appropriate neuromodulation [44, 45]. Studying the progression of brain function in patients receiving regular antipsychotics after clinical diagnosis is therefore necessary to develop antipsychotics and neuromodulation technologies.

Using an innovative dynamic analysis framework for multiple FIs in the present study, we identified five progressive stages of schizophrenia in patients after diagnosis. Consistent with prior studies, a trajectory of clinical symptoms was identified from predominantly positive to predominantly negative symptoms, with five distinct stages that were classified as positive-dominated, negative ascendant, negative-dominated, positive ascendant, and negative surpassed. A hypofunction trajectory also emerged from the primary sensorimotor and visual cortices to the salience system and then to the default mode network, which contributes to abnormal external sensory gating in schizophrenia. Furthermore, a hyperfunction trajectory was identified from subcortical regions to the hippocampus and then to the dorsal frontoparietal network, which is associated with a disrupted internal excitation–inhibition equilibrium. We also identified that the regions located in primary and salience systems were strongly associated with behavior in patients at relatively early stages, whereas regions located in higher-order and subcortical systems were significantly associated with behavior in the later stages of disease progression. The trajectories of progressive dysfunction were nonlinearly associated with cerebral gradients, which were specifically concordant with the trajectories of positive symptoms. These findings further indicate that the dysfunction identified in the present study is essentially the same as that of previously reported cerebral gradients, and reflects integrated brain functional profiles. Furthermore, dysfunction-associated genetic analysis revealed that neurodevelopmental and neurodegenerative factors were associated with specific patterns of dysfunction over the disease progression and specifically highlighted the dopamine-centered synaptic system in the early disease stages. Together, these findings indicate the existence of progressive neuroimaging dysfunction with associated genetic factors in schizophrenia and will be useful for establishing an integrated framework to better understand the pathomechanisms of this disease.

A previously proposed clinical course of schizophrenia defined three representative phases (treatment, relapse, and chronic phases) after first-episode psychosis [7]. In the treatment phase, positive symptoms are well-controlled, with a sharp slope, and negative symptoms present a gradual slope. Next, both the positive and negative symptoms ascend as the disease progresses (with a greater slope observed in positive symptoms). This is followed by a descent into the chronic phase, characterized by predominantly negative symptoms. Similarly, our work identified five stages—from predominantly positive to predominantly negative symptoms—after first-episode psychosis; this is largely consistent with the recognized clinical course of schizophrenia and supports the rationality of our analysis framework. Furthermore, in line with the conventionally defined stages of relapse, we characterized dynamic changes in the dominance of positive and negative symptoms. Symptoms from the positive-dominated, negative-ascendant, and negative-dominated stages correspond to the phase that is well-controlled with antipsychotics [46]. The positive-ascendant stage corresponds to the relapse phase, and the negative-surpassed stage indicates the beginning of the chronic phase [47, 48].

Consistent with previous meta-analyses of FIs [49], the results of the current study suggest that hypofunction occurs in the primary sensorimotor and visual cortices in the early stage of schizophrenia. Previous studies have documented sensory and perceptual deficits in early-stage processing and cognitive behavior [50] that are associated with specific clinical symptoms, such as delusions, hallucinations, and decreased voluntary motion. In the present study, decreased FIs were observed in lower-order regions (involving the primary sensorimotor and visual cortices) in this positive-dominated stage, which are associated with a potential mechanism for self-disorder in schizophrenia [51] and suggest a disconnect with real external stimuli in the world [52]. Significant associations between drug equivalents and FIs were also observed in visual cortices in the present study; this finding may be related to the response of positive symptoms to antipsychotics. There is substantial evidence of a hyperresponsive dopamine system in schizophrenia, and striatal dopamine pathways are strongly involved in disease progression [7]. It is therefore unsurprising that we identified striatal hyperfunction in all disease stages in the present study; this finding is in line with the recognized hyperresponsive dopamine system in schizophrenia. Furthermore, abnormal striatal dopamine synthesis is a specific feature of the prodromal stage and worsens as the disease progresses [53, 54]. The patterns of dysfunction were significantly spatially correlated with the distribution of striatal dopamine synthesis, release capacity, and transporters in the current study; these results further support the fundamental role of striatal dopamine pathways in the progression of schizophrenia. Striatal hyperfunction and hypofunction in lower-order regions might therefore be representative phenotypes of functional disturbance in the early positive-dominated phase of disease progression after clinical diagnosis.

The blockade of dopamine receptors can improve clinical symptoms in patients with schizophrenia, suggesting a common disruption of dopaminergic pathways [55]. A hyperresponsive dopamine system in schizophrenia has been suggested to result from a tendency of the brain to overrespond to external salient stimuli, independent of their importance, thus allocating disturbance salience when processing signals [56]. Similarly, the sensory gating hypothesis suggests that the pathological basis of schizophrenia is the absence of sensory gating, which leads to a large amount of irrelevant information entering the brain and disrupting its function [57]. The salience network is involved in the process of differentiating relevant from irrelevant stimuli and assigning salience to stimuli-focused information [58]. In schizophrenia, however, the salience process is disrupted by aberrant dopamine signaling related to irrelevant stimuli [59].

In the present study, as the disease progressed, we identified hypofunction in the insula. This finding supports the theory of a disrupted salience-monitoring system in schizophrenia. Furthermore, insular hypofunction was accompanied by hypofunction of the default mode network, which is associated with abnormalities of ongoing information processing (including of inner reference, memory, and emotions) in schizophrenia [60]. An abnormal functional interaction among the so-called triple network (the salience, default mode, and central executive networks) has been proposed to explain the pathophysiological dysfunction underlying psychiatric disorders [61], and as a marker to understand the vulnerability of external and internal perceptions in patients with schizophrenia [62]. The interaction between the salience and default mode networks has been suggested to be strongly linked to positive symptoms, but accumulating evidence also indicates that a complex association exists between triple network profiles and positive, negative, and cognitive symptoms in schizophrenia [63]. Consistent with those of sensory gating, these findings might partially explain the cascade of impairments from lower- to higher-level functions in patients with schizophrenia.

In the present study, the orbitofrontal cortices worsened as the disease progressed. The orbitofrontal cortices play important roles in value processing and positive affect; their abnormal function might therefore induce apathy and lack of effect, which are typical clinical symptoms of schizophrenia. Thus, our findings are in line with the recognized aberrant motivation and reward-based learning that occur in schizophrenia [64].

As the disease progresses, negative symptoms ascend to be dominant after a phase of well-controlled symptoms. In the current study, marked hyperfunction was observed in the thalamus, cerebellum, and hippocampus as the disease progressed. The striatal–thalamocortical network is important for integrating complex information and has been associated with cognitive and emotional deficits in patients with schizophrenia [65]. Notably, the dopaminergic system is not the only aberrant system in schizophrenia; its modulatory γ-aminobutyric acid (GABA)ergic and glutamatergic synaptic systems also contribute to the disease. The hippocampus plays a crucial role in driving the dopaminergic system and contributes to the underlying pathophysiology of schizophrenia [2, 66, 67]. Increased hippocampal glutamate function is associated with increased striatal dopamine function in schizophrenia, as well as with dysfunction across symptom domains [67]. Hippocampal hyperfunction might therefore be a neuroimaging phenotype that implies long-term memory impairment in schizophrenia [66]. From a molecular perspective, a magnetic resonance spectroscopy study reported that higher GABA concentrations in the cerebellum are associated with greater behavioral impairments in patients with schizophrenia. Increased cerebellar function gradually emerges as the disease progresses, implying that cerebellar GABAergic modulation plays a role in schizophrenia. An excitatory–inhibitory imbalance in the cerebello-thalamo-cortical and striato-thalamo-cortical loops has been proposed to explain the pathology and development of schizophrenia, in which the dopamine and GABA systems make predominant contributions [7, 68, 69]. The findings of the present study also suggest that the striatum and cerebellum may predominantly contribute to the progressive excitatory–inhibitory disruption, which is in line with the “cognitive dysmetria” theory of schizophrenia [70]. In general, the trajectory of hyperfunction might reflect the progressive dysfunction of the dopaminergic system and its upstream GABA and glutamate modulator systems in patients with schizophrenia, which may correspond to their complex clinical symptoms.

Genome-wide association studies have identified more than 50 genes associated with the pathology of schizophrenia, which are mainly implicated in neurodevelopment and the dopamine-centered synaptic system [71]. In the current study, specific genetic features were identified in duration-labeled progressive stages. Multiple factors relating to neurodevelopment were revealed as the disease progressed, including *AH1* and the phosphoinositide 3-kinase–protein kinase B signaling pathways. Dopaminergic, glutamatergic, and GABAergic synapses were markedly enriched as early risk factors in the positive-dominated stage, thus supporting a substrate role of the dopaminergic synapse, which has been widely recognized as a cause of schizophrenia [72]. Our work thus provides explicit evidence to support the early involvement of dopaminergic synapses and highlights the role of the excito-inhibitory synaptic system as the disease progresses. The glutamatergic and GABA pathways are crucial in the upstream regulation of dopaminergic function [73], and their interaction contributes to the overall dysfunction that occurs in schizophrenia [74]. It has been proposed that imbalanced inhibitory and excitatory signaling complexes are a pathomechanism of schizophrenia [75]. These interconnected synaptic systems regulate brain plasticity and are a potential intrinsic cause of schizophrenia, as well as being associated with the complex symptoms of patients [76, 77].

Another notable finding of the present study was the emergence of neurodegenerative factors in relatively late stages of disease progression. A neurodegenerative model has been proposed to understand the mental decline that occurs in schizophrenia, along with its progressive clinical course [78]. Although postmortem examinations have not found neurodegenerative pathological features (such as gliosis) in schizophrenia [79], the neurodegenerative model suggests that pathological neuronal apoptosis (which does not cause gliosis) occurs in schizophrenia, whereas necrosis does not. In support of this model, apoptosis-related genetic factors were identified in the present study. Furthermore, a progressive neurodevelopmental hypothesis has redefined the boundaries of neurodevelopment and neurodegeneration in schizophrenia, and views schizophrenia as having components of both development and degeneration [5, 80]. Although the present findings do not directly indicate that schizophrenia is a neurodegenerative disease, they do suggest that neurodegeneration-related genes might be involved later in the disease. Thus, the neurodegenerative hypothesis of schizophrenia requires further investigation.

### Limitations

There are several limitations of the present study. First, our approach has high requirements for data. The sample size needs to be relatively large and the disease course distribution of patients needs to be relatively uniform, which may limit its application. Although we validated the associated genes in the non-progressive analysis using a replication cohort from the UCLA, we were unable to identify a suitable dataset to validate the progressive analysis. Moreover, although the drug equivalent represents the stable drug intake of patients within the last year of data collection, it only provides a partial possible drug effect. The drug effects in this study should therefore be analyzed using more powerful models. We sought to explore the association between neuroimaging and the transcriptome in patients with schizophrenia in the present study. The best way to do this would be to directly link each patient’s neuroimaging data to their transcriptome data. Unfortunately, however, transcriptome data were not available from the patients with neuroimaging data. A compromise was therefore made; the case–control differences in neuroimaging were linked to healthy transcriptome data from the AHBA database, to indirectly identify genes that are associated with the disease. This association analysis of the brain imaging data of disease samples and the gene expression levels of healthy samples was likely affected by sample heterogeneity, which should be improved in future studies (e.g., by using genetic data from disease samples). Moreover, the association among neuroimaging, genetic features, and behavior was characterized by the PLSR model. This model can be overfitted and is not stable across datasets, especially when the number of samples is small; these limitations severely hinder the interpretability and generalizability of the results [81]. Therefore, a further validation analysis with larger data samples is needed.

## Conclusions

To investigate the stage-specific patterns of dysfunction after diagnosis of schizophrenia, we built a novel analytical framework to explore duration-labeled progressive stages using cross-sectional data. A refined symptom trajectory with five stages was recognized. Furthermore, we identified a progressive hypofunction trajectory from primary lower- to higher-level cortices and a progressive hyperfunction trajectory from subcortical to higher-level cortices, which are associated with abnormal external sensory gating and disrupted internal excitation–inhibition equilibrium in schizophrenia. Progressive dysfunction correlated with specific symptoms across the different stages. By combining our data with genetic data from independent healthy controls, we further identified a dopaminergic-centered synaptic system in the early stages of schizophrenia, and neurodegenerative factors in the later stages. Additionally, substrates of neurodevelopment carried risks for disease progression. Together, these findings suggest that our proposed innovative analytical framework has the potential to reveal the genetic factors associated with progressive functionalities in schizophrenia, thus inferring the dynamic involvement of gene endophenotypes associated with clinical phenotypes.

## Supplementary Information

**Additional file 1: Fig. S1.** Analysis flowchart. **Fig. S2.** Flow chart of the alignment across states of FIs.

**Additional file 2: Table S1.** Subgroup information with a window length of 5 years. **Table S2.** Subgroup information with a window length of 6 years. **Table S3.** Subgroup information with a window length of 7 years. **Fig. S3.** PANSS positive, negative, general, and total scores in all subgroups. **Fig. S4.** Calinski–Harabaz index in the different number of clusters with APC. **Fig. S5.** Explained variance and variance gain in the *k*-means approach. **Fig. S6.** APC results and alignment with different window lengths. **Fig. S7.** Stage patterns with window lengths of 6 and 7 years. **Fig. S8.** Correlation between progressive dysfunction and cerebral function gradients. **Fig. S9. **Neuroimaging PLS1 loadings across stages. **Fig. S10.** Relationship between disease duration and drug equivalent. **Fig. S11.** Correlation between FIs and drug equivalent. **Fig. S12.** Comparison of FIs between patients with high and low drug equivalents. **Fig. S13.** Relationship between disease duration and brain structural features. **Fig. S14.** Spatial correlation between case–control *t*-maps of five functional indicators and dopamine synthesis. **Fig. S15.** Spatial correlation between case–control *z*-maps of five stages and dopamine synthesis. **Fig. S16.** Correlation between PLS1 maps and case–control* t*-maps in all progressive stages of the disease. **Fig. S17.** Enriched terms across stages and PLS1gene lists, colored by *p*-values. **Fig. S18.** KEGG enrichment network from merged PLS1+genes of the five progressive stages.

**Additional file 3:** **Fig. S19.** Case–control *t*-maps of functional indicators and their spatial associations with PLS1. **Fig. S20.** Genes related to the case–control *t*-maps of the five functional indicators. **Fig. S21.** KEGG networks using merged PLS1 genes. **Fig. S22.** Case–control *t*-maps and PLS1 maps of FIs in the discovery and replication datasets. **Fig. S23.** KEGG networks using merged PLS1 genes.

## Data Availability

Data availability: Molecular architecture (density maps of neurotransmitter receptors and transporters) for the integration of positron emission tomography and single-photon emission computed tomography from the prior vivo molecular imaging studies are available at https://github.com/netneurolab/hansen_receptors/tree/main/data and https://github.com/juryxy/JuSpace. Human brain-wide gene expression samples from the Allen Human Brain Atlas are available at https://human.brain-map.org/static/download. Schizophrenia disease-related genes (*N* = 52) from in situ hybridization are available at https://human.brain-map.org/ish/search. All data generated or analyzed during the current study are included in the published article (and its Additional file 1: Supplementary Information). Code availability: The DPABI and NIT toolbox for FI calculation is available at http://www.rfmri.org/dpabi and https://www.neuro.uestc.edu.cn/NIT.html [26, 82]. The code for gene expression analysis can be found at https://github.com/BMHLab/AHBAprocessing [37]. The code for PLSR analysis used in this study can be found at https://github.com/jssuestc/MultimodalCorr, which is written based on https://github.com/SarahMorgan/Morphometric_Similarity_SZ [18], https://github.com/spin-test/spin-test, and https://github.com/frantisekvasa/rotate_parcellation [39]. The ENIGMA toolbox helps the codes for the spin test in this work (v. 1.1.3; https://enigma-toolbox.readthedocs.io/en/latest/) [83]. The Metascape tool of gene enrichment analysis (version 3.0) was available at http://metascape.org/.

## Abbreviations

MRI: Magnetic resonance imaging
FIs: Functional indicators
AHBA: Allen Human Brain Atlas
PANSS: Positive And Negative Syndrome Scale
PLSR: Partial linear squares regression
KEGG: Kyoto Encyclopedia of Genes and Genomes
PET: Positron emission tomography
SPECT: Single-photon emission computerized tomography
FDR: False discovery rate
MCODE: Molecular Complex Detection
