# Supplementary material for: Progressive trajectories of schizophrenia across symptoms, genes, and the brain
Source: BMC Med. 2023 Jul 3;21:237. doi: 10.1186/s12916-023-02935-2 (PMC10318676; doi:10.1186/s12916-023-02935-2)
Supplement: Supplementary file 1 — Additional file 1: Fig. S1. Analysis flowchart. Fig. S2. Flow chart of the alignment across states of FIs. [file 12916_2023_2935_MOESM1_ESM.docx]

***Supplementary materials for “Progressive trajectories of schizophrenia across symptoms, genes, and the brain*”**


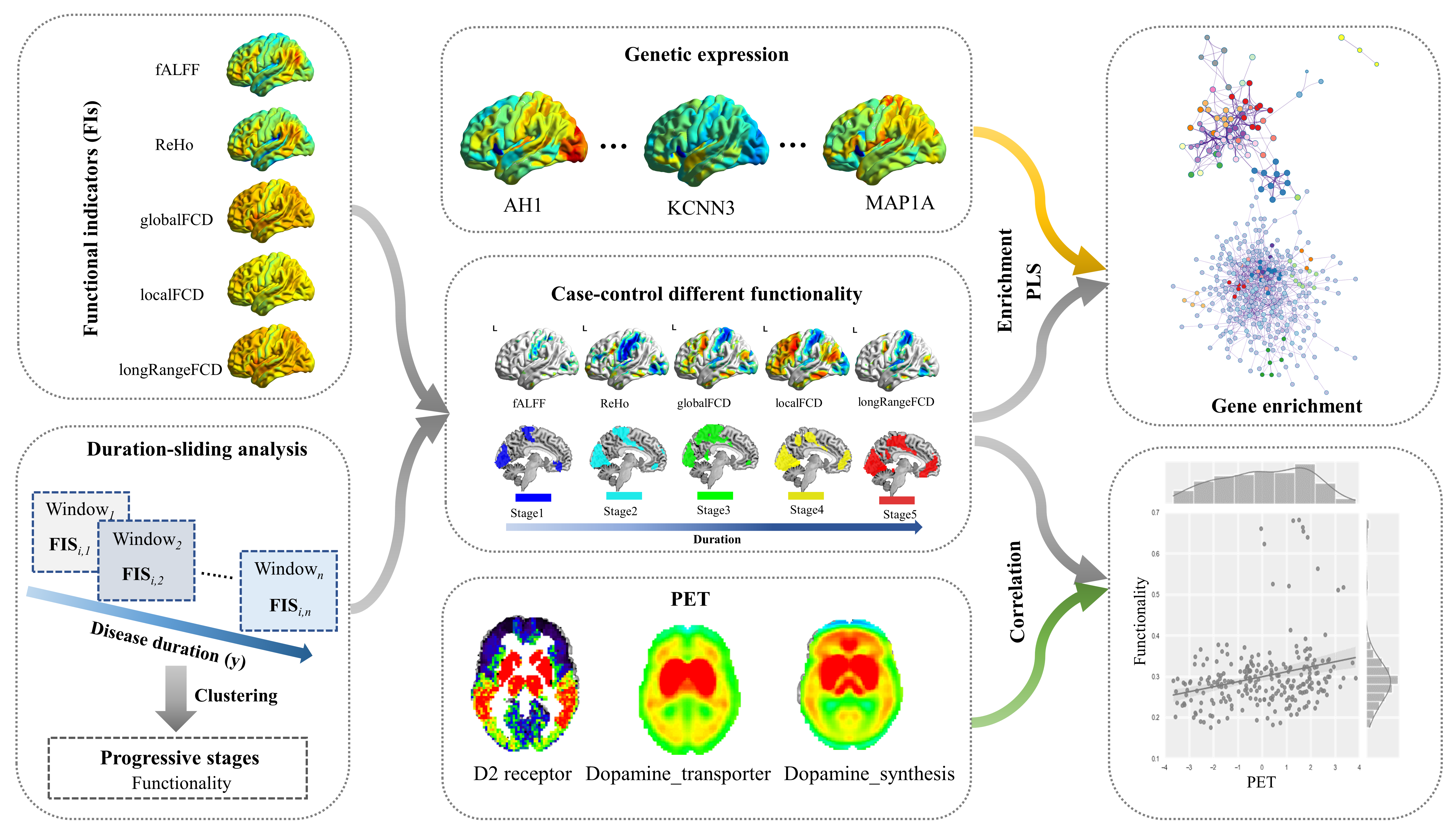


**Fig. S1.** Analysis flowchart. Five classical functional indicators (FIs) were selected to measure brain functionality. FIs were calculated in a set of subgroups generated by a duration-sliding approach and further clustered into duration-labeled progressive stages with specific neuroimaging profiles. Association analyses among neuroimaging, gene, and metabolism features were further performed, resulting in a framework across multiple models.

# Method S1. Data preprocessing and FIs calculation

The first five volumes of each run were discarded to eliminate magnetic field instability. Using a home-made Neuroscience Information Toolbox, slice-timing correction, realignment, and spatial normalization to the Montreal Neurological Institute (MNI) template were conducted successively. Then, these images were resampled to an isometric 3x3x3 mm3 grid. We excluded the subjects with head motion exceeding 2 mm or/and 2 degrees. Besides, 24 head-motion parameters, white matter signals, cerebrospinal fluid signals, and the signal of the whole brain were regressed from normalized data. Computational Anatomy Toolbox (<http://www.neuro.uni-jena.de/cat/>) was used to estimate the grey matter volume, white matter volume, and cerebrospinal fluid volume.

*The fractional amplitude of low-frequency fluctuation (fALFF)*: Normalized functional images were smoothed using an isotropic Gaussian filter kernel with full width at half maximum (FWHM) of 8mm. The time series for each voxel was transformed to the frequency domain and the power spectrum was then obtained. Since the power of a given frequency is proportional to the square of the amplitude of this frequency component, the square root was calculated at each frequency of the power spectrum. The sum of amplitude across 0.01–0.08 Hz was divided by that across the entire frequency range (0–0.25 Hz).

*Regional homogeneity (ReHo)*: A temporal filter in the 0.01–0.1 Hz band was performed in the normalized functional images. No spatial smoothing was performed to avoid introducing the artificial local spatial correlation. The regional homogeneity of a given voxel was assigned by calculating the Kendall coefficient of concordance of the time series of this voxel with those of its 26 nearest neighbors.

*Functional connectivity density (FCD)*: Pearson’s correlation coefficients were computed between processed time courses of all voxels of the brain. In this study, a correlation threshold of 0.6 was used to generate binary connectivity. The global FCD of a given voxel is the number of significant connectivity between that voxel and all voxels in the whole brain. The local FCD is defined by the number of functional connectivity between a given voxel and its direct and indirect neighbor voxels. The long-range FCD is equal to global FCD minus local FCD. In this study, the significant level is defined as correlation coefficients larger than 0.6.

# Method S2. Case-control statistics of FIs

All FIs were z-scored for normalization. One-sample t-tests were used to investigate within-group statistical t-maps, generating masks with a threshold of p<0.001. Case-control differences were detected by two-sample t-tests (p<0.05, FDR corrected). Notably, case-control differences were restricted in a union mask of one-sample t-tests of both groups. The duration is positively correlated with the age of patients (r(80)=0.66, p<0.001). Since the age didn’t match between patients and controls, to reduce the influence of age on the results, we regressed out age, gender, FD, and intracranial volume in the statistics.

# Method S3. Affinity propagation clustering

For each subgroup, multiple FIs were calculated and compared with the HC group using two-sample t-tests, generating a series of case-control t-maps (without threshold) for each functional feature.

Affinity propagation clustering (APC) performs well in recognizing face images, identifying representative sentences, and detecting genes. Besides, APC overcomes the shortcomings of K-means which is sensitive to the initial exemplar and needs a pre-specified number of clusters. APC algorithm employed responsibility and availability to access information exchange between data points. Preference at the beginning and a dumping factor in the iteration are two parameters of APC. An exemplar can be recognized by maximum the summation of responsibilities and availabilities. Iterations would be terminated when reaching a specified number of iterations, faint increments of information change, or decisions staying constant for a certain number of iterations.In this study, Euclidean distance was used to measure the similarity between statistic maps in different windows, the preference was set to the median of the similarity, and the dumping factor was set to 0.5. That is, 5 times affinity propagation clustering was carried out in the current study, generating a set of functional states characterized by case-control t-maps in each indicator.

To obtain different numbers of clusters, we fixed the dumping factor to 0.5 and varied the value of preference in the range of 0.9 to 1.1 times the median of similarity in steps of 0.01.

To select an appropriate number of clusters, we further accessed the Calinski-Harabaz index (CHI) in different cluster numbers. A higher CHI represents greater clustering.

For a given state in one of the FIs, it was labeled with a different range of disease duration depending on the sliding window it contains. For instance, if state 1 of fALFF contains the first three windows, then the range, mean, and standard deviation of the duration of state 1 were determined by the duration of all subjects in the first three windows.

# Method S4. Alignment across states of FIs

As shown below, a flow chart illustrates the procedure of alignment across states of FIs obtained from APC (Fig. S2). The spatial similarity (ranging from 0-1) was estimated by the Dice coefficient between spatial patterns of stages (similarity between significant case-control z-maps). The temporal overlap (ranging from 0-1) was defined by the Dice coefficient of the duration range between states. We constructed the product (ranging from 0-1) of spatial similarity and temporal overlap to provide an integrated measurement accessing the similarity between states from different FIs. A higher value of the product indicates higher similarity. Thus, states from different FSI maximizing the product were gathered together into a stage. By fully merging states from all indicators, five states were obtained with labeled disease duration. We provide a flow chart and formulas to demonstrate this below.

$${SS}_{i,j}=\frac{NV\left( S_{i}\cap S_{j} \right)}{NV\left( S_{i}\cup S_{j} \right)}{; TS}_{i,j}=\frac{{RD}_{i}\cap{RD}_{j}}{{RD}_{i}\cup{RD}_{j}}$$

$${IS}_{i,j}={SS}_{i,j}\times{TS}_{i,j}$$

The ${SS}_{i,j}$ indicates the spatial similarity between the $i_{th}$ and $j_{th}$ state from different FIs. The $S_{i}$ and $S_{j}$ indicate the statistical map (p<0.05, FDR corrected) of the $i_{th}$ and $j_{th}$ state from different FIs and the *i* and *j* can be equal. The $NV\left( S_{i}\cap S_{j} \right)$ indicates the number of voxels at the intersection of $S_{i}$ and $S_{j}$. The ${TS}_{i,j}$ indicates the temporal similarity between the $i_{th}$ and $j_{th}$ state from different FIs. The ${RD}_{i}$ and ${RD}_{j}$ indicate the range of disease duration of the $i_{th}$ and $j_{th}$ state from different FIs and the *i* and *j* can be equal. The ${IS}_{i,j}$ indicates the integrated similarity between the $i_{th}$ and $j_{th}$ state from different FIs. Similar to the previous rules, the final stage contains distinct states and with it a corresponding range, mean, and standard deviation of disease duration.


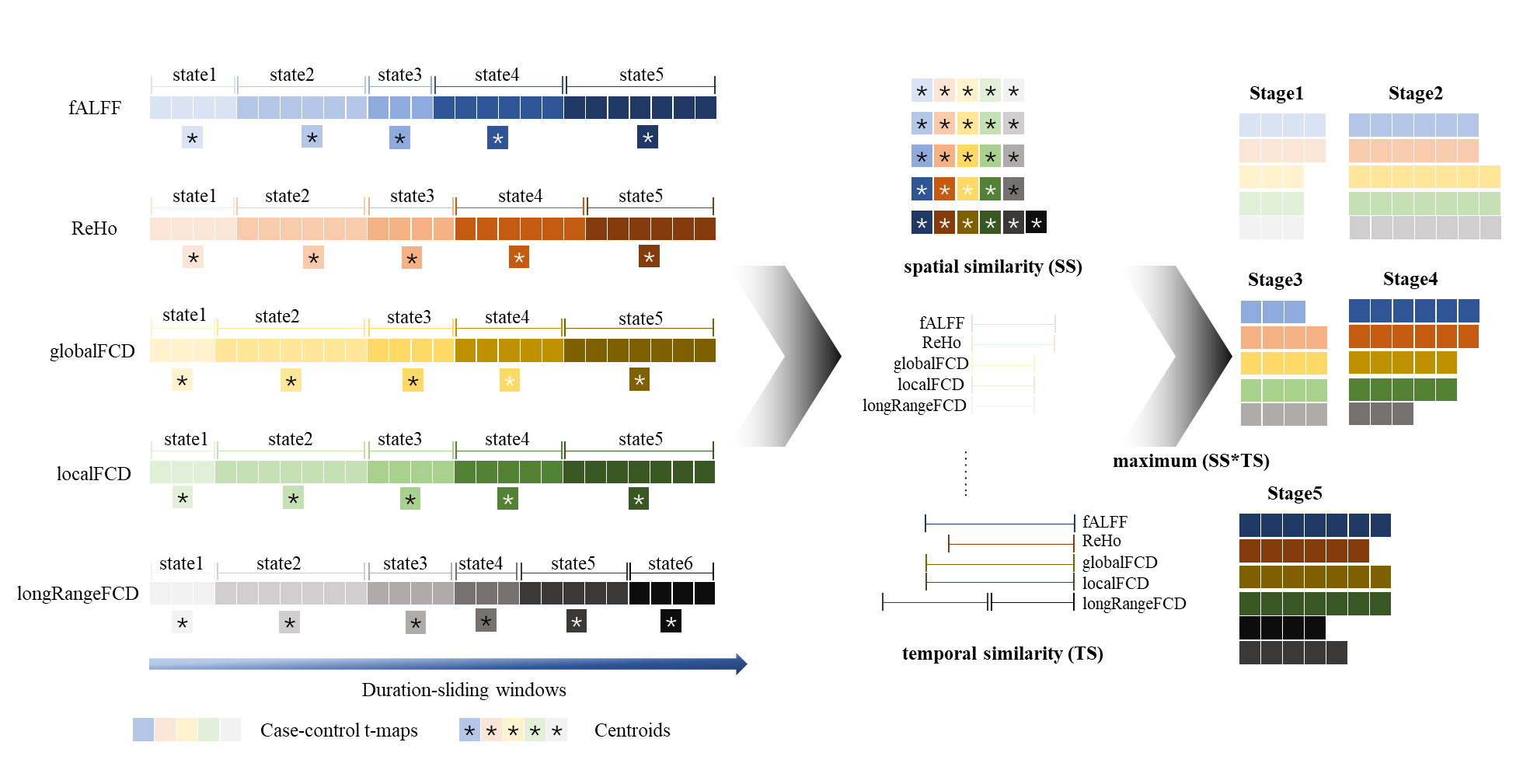


**Fig. S2.** Flow chart of the alignment across states of FIs. The APC approach was first conducted in case-control t-maps of sliding windows in each of the FIs, resulting in series clustering states. Subsequently, spatial similarity (SS) and temporal similarity (TS) between all pairs of states across FIs. The final stages were determined by the combination of FIs states maximizing the product of SS and TS.

# Method S5. Dysfunction patterns of progressive stages

Each specific disease stage was determined by states coming from distinct functional states, defined by clustering subgroup case-control t-maps. The Liptak–Stouffer formula was used to obtain the integrated case-control z-maps to reveal the final dysfunction of duration-specific stages in schizophrenia patients. Specifically, the case-control t-maps in each subgroup were converted to z-maps for standardization using the inverse normal distribution function. Then, combined z-scores integrating results from different subgroups were obtained using the Liptak–Stouffer formula :

$$Z=\frac{\sum_{i=1}^{k} w_{i}z_{i}}{\sqrt{\sum_{i=1}^{k} w_{i}^{2}}}$$

where *W_i_* is the square root of the number of the *i*th subgroup. Finally, the combined Z values were transformed into their corresponding p-values. The threshold was set at p-value <0.05 with family discovery rate (FDR) correction to display between-group differences.

# Method S6. Genome expression data

The Allen Human Brain Atlas (AHBA) database (http://human.brain-map.org) provides gene expression of six healthy adult human donors (1 female and 5 male, 24-57 years old). Six major steps were performed to generate regional gene expression, including verifying probe-to-gene annotations (45,812 probes were uniquely annotated, corresponding to 20,232 unique genes), filtering probes do not exceed background noise, selecting probes index expression for a gene, mapping tissue samples to specific brain regions, normalizing expression to account for inter-individual differences and outlying values, selecting genes consistently expressing across six brains. Finally, 10,027 genes surpassing quality-control criteria were obtained. In this work, tissue samples were assigned to an atlas with 246 regions of cerebral cortices. Since the AHBA only included two right hemispheres in two donors, this study only used the left hemisphere data. Thus, we obtained expression values for each of the 10027 genes estimated in 123 fMRI regions, that is a 123×10027 regional transcription matrix.

# Method S7. Dysfunction-associated genes

Statistical maps were divided into 246 regions based on an atlas provided by Fan et al., with values extracted for 123 regions in the left hemisphere. In the partial linear squares regression (PLSR) model, dysfunction characteristics (statistic t values) of progressive stages were predicted by the whole brain genome expression. Linear combinations of gene expression were revealed to link to dysfunction characteristics. The PLSR identified a set of genes (dysfunction-associated genes) significantly correlating to the case-control comparisons.

According to the “Genes characterized by ISH in 1,000 gene survey in the cortex (Cortex Study)” provided by the Allen Human Brain Atlas, fifty-two schizophrenia genes were recognized. In this study, we overlapped the dysfunction-associated genes and fifty-two recognized schizophrenia-related genes. Then, the expression of the overlapped genes in the whole brain was correlated to the statistical t-values or z-values. An FDR-corrected p<0.05 was used to detect significant correlations.

# Method S8. Non-progressive analysis

Regular non-progressive case-control comparisons were also performed in this work. In the discovery cohort, after FIs calculation in all participants in the discovery cohort, all schizophrenia patients were gathered into one group and compared with the controls using the two-sample t-test. Significant case-control differences were detected with p<0.05 with FDR correction. Dysfunction-associated genes and enrichment analysis were conducted using the same method as in the progressive analysis. Multiple gene lists were generated by merging genes associated with the case-control t-maps of all FIs.

# Method S9. Validation analyses

## k-means clustering

A traditional k-means clustering approach was additionally performed to further validate the selection of the optimal cluster number. We calculated the variance explained by clustering data with k=2-25. Percentage gain in variance explained when advancing from k-1 to k. The explained variance is defined by the ratio of the between-cluster variance and the total variance. The total variance equals the within-cluster plus the between-cluster variance. The within-cluster variance was computed as the clusters-averaged sum of square distances between data points in a cluster and its centroid. The between-cluster variance was computed as the average square distance between a cluster centroid and the centroid of all clusters or centroid of all data.

## A replication cohort

Fifty schizophrenia patients (38 males, age 36.46±8.88) and ninety-three healthy controls (49 males, age 30.14±8.20) were recruited from the UCLA data set. Five functional characteristics were calculated and compared with the HC, and further related to genes. Based on a replication cohort from UCLA, we performed case-control differences of FIs, identified dysfunction-associated genes, and enrichment analyses.

## Window length

To test the reliability of progressive functionality trajectory, we recalculate the FIs and clustering analyses with a window length of 6 years and 7 years.

# Method S10. Control analyses

## Morphological characteristics of the brain

Correlation between the illness duration and brain structural features (including grey matter volume, white matter volume, and cerebrospinal fluid volume), with age and gender as nuisance covariates. First, we sorted the patients in ascending order of disease duration. Then, we divided patients into two subgroups, including a subgroup of 20% of patients with short duration, and a subgroup of 20% of patients with long duration.

## Antipsychotics

However, we still hope to find a little bit of a relationship between the effects of drugs on the brain neuroimage. First, we compared the drug equivalent between stages using a permutation test. All patients were randomly divided into two groups and the average drug equivalent was compared between groups. A null model was constructed by performing 1000 random grouping and drug equivalent comparisons.

Besides, we further sorted patients according to the drug equivalent and compared the FIs between patients with high and low drug equivalents. We compared the FIs of patients in the top 20/30 percent of drug equivalents with those in the bottom 20/30 percent.
