# Supplementary material for: Progressive trajectories of schizophrenia across symptoms, genes, and the brain
Source: BMC Med. 2023 Jul 3;21:237. doi: 10.1186/s12916-023-02935-2 (PMC10318676; doi:10.1186/s12916-023-02935-2)
Supplement: Supplementary file 2 — Additional file 2: Table S1. Subgroup information with a window length of 5 years. Table S2. Subgroup information with a window length of 6 years. Table S3. Subgroup information with a window length of 7 years. Fig. S3. PANSS positive, negative, general, and total scores in all subgroups. Fig. S4. Calinski–Harabaz index in the different number of clusters with APC. Fig. S5. Explained variance and variance gain in the k-means approach. Fig. S6. APC results and alignment with different window lengths. Fig. S7. Stage patterns with window lengths of 6 and 7 years. Fig. S8. Correlation between progressive dysfunction and cerebral function gradients. Fig. S9. Neuroimaging PLS1 loadings across stages. Fig. S10. Relationship between disease duration and drug equivalent. Fig. S11. Correlation between FIs and drug equivalent. Fig. S12. Comparison of FIs between patients with high and low drug equivalents. Fig. S13. Relationship between disease duration and brain structural features. Fig. S14. Spatial correlation between case–control t-maps of five functional indicators and dopamine synthesis. Fig. S15. Spatial correlation between case–control z-maps of five stages and dopamine synthesis. Fig. S16. Correlation between PLS1 maps and case–control t-maps in all progressive stages of the disease. Fig. S17. Enriched terms across stages and PLS1gene lists, colored by p-values. Fig. S18. KEGG enrichment network from merged PLS1+genes of the five progressive stages. [file 12916_2023_2935_MOESM2_ESM.docx]

***Supplementary materials for “Progressive trajectories of schizophrenia across symptoms, genes, and the brain*”**

# Results

# Duration-sliding subgroups in schizophrenia

Specifically, the first subgroup had a disease duration of 0 to 5 years, the second subgroup had a disease duration of 1 to 6 years, and so on.

Table S1. Subgroup information with a window length of 5 years.

| subgroup | Duration range(year) | Mean duration | Number |
| --- | --- | --- | --- |
| subgroup_1 | 0-5 | 2.274 | 19 |
| subgroup_2 | 1-6 | 2.84 | 17 |
| subgroup_3 | 2-7 | 4.24 | 17 |
| subgroup_4 | 3-8 | 5.29 | 17 |
| subgroup_5 | 4-9 | 6.71 | 17 |
| subgroup_6 | 5-10 | 8.38 | 16 |
| subgroup_7 | 6-11 | 8.79 | 19 |
| subgroup_8 | 7-12 | 8.94 | 18 |
| subgroup_9 | 8-13 | 9.94 | 16 |
| subgroup_10 | 9-14 | 10.64 | 14 |
| subgroup_11 | 10-15 | 12.15 | 13 |
| subgroup_12 | 11-16 | 13.83 | 12 |
| subgroup_13 | 12-17 | 15 | 10 |
| subgroup_14 | 13-18 | 15.69 | 13 |
| subgroup_15 | 14-19 | 16.93 | 15 |
| subgroup_16 | 15-20 | 17.78 | 18 |
| subgroup_17 | 16-21 | 18.5 | 16 |
| subgroup_18 | 17-22 | 19.76 | 17 |
| subgroup_19 | 18-23 | 20.42 | 19 |
| subgroup_20 | 19-24 | 21.22 | 18 |
| subgroup_21 | 20-25 | 22.25 | 16 |
| subgroup_22 | 21-26 | 23.43 | 14 |
| subgroup_23 | 22-27 | 24.25 | 16 |
| subgroup_24 | 23-28 | 25.23 | 13 |
| subgroup_25 | 24-29 | 26.42 | 12 |
| subgroup_26 | 25-30 | 26.9 | 10 |

# PANSS scores of subgroups

Each subgroup has recorded PANSS scores, including positive, negative general, and total scores. The below curves illustrate subgroup-level scores (Fig.. S3).

**
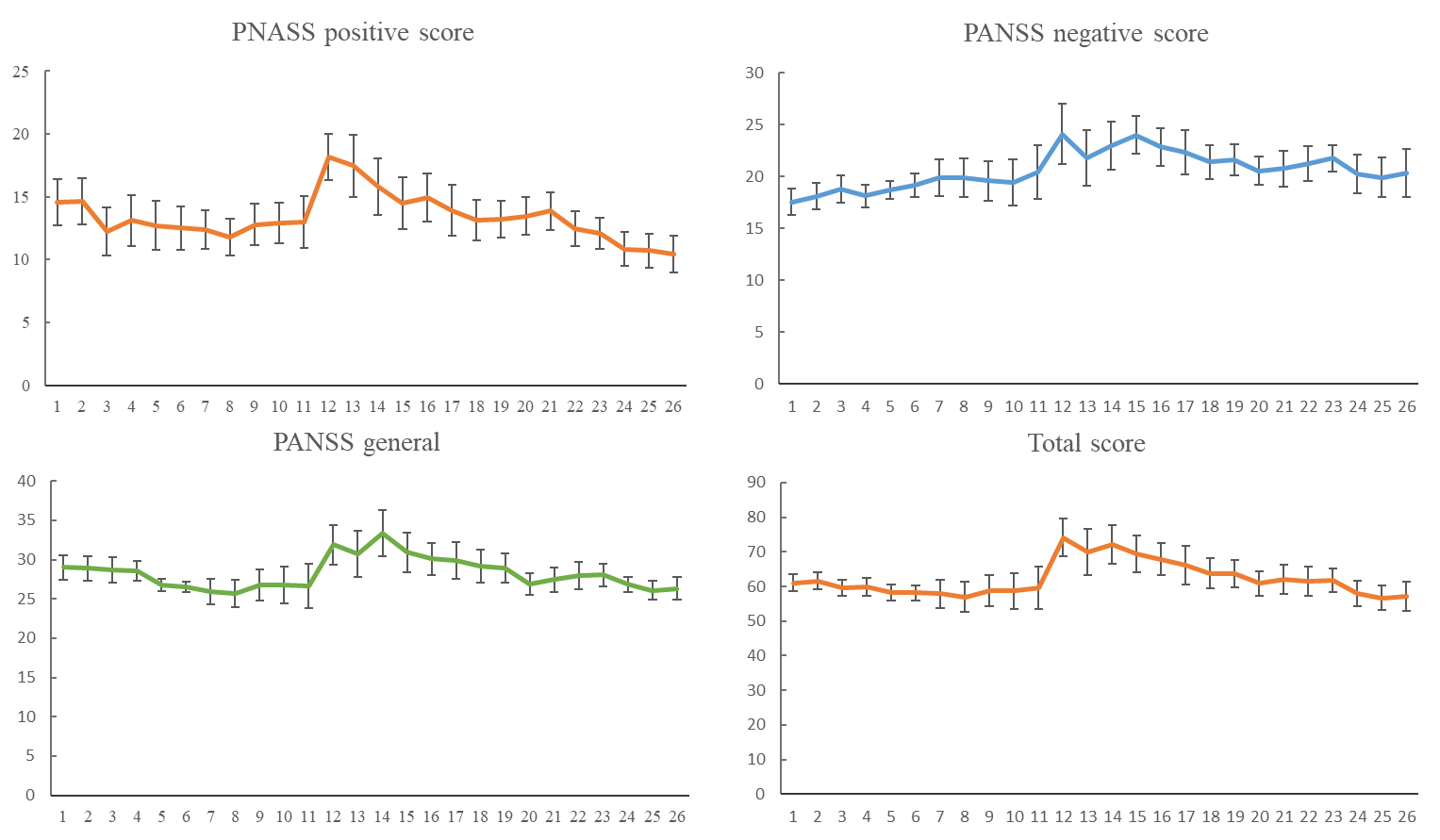
**

**Fig. S3.** PANSS positive, negative, general, and total scores in all subgroups. The curves represent mean scores in each subgroup and the error bars represent the standard error.

# The optimal number of clustering


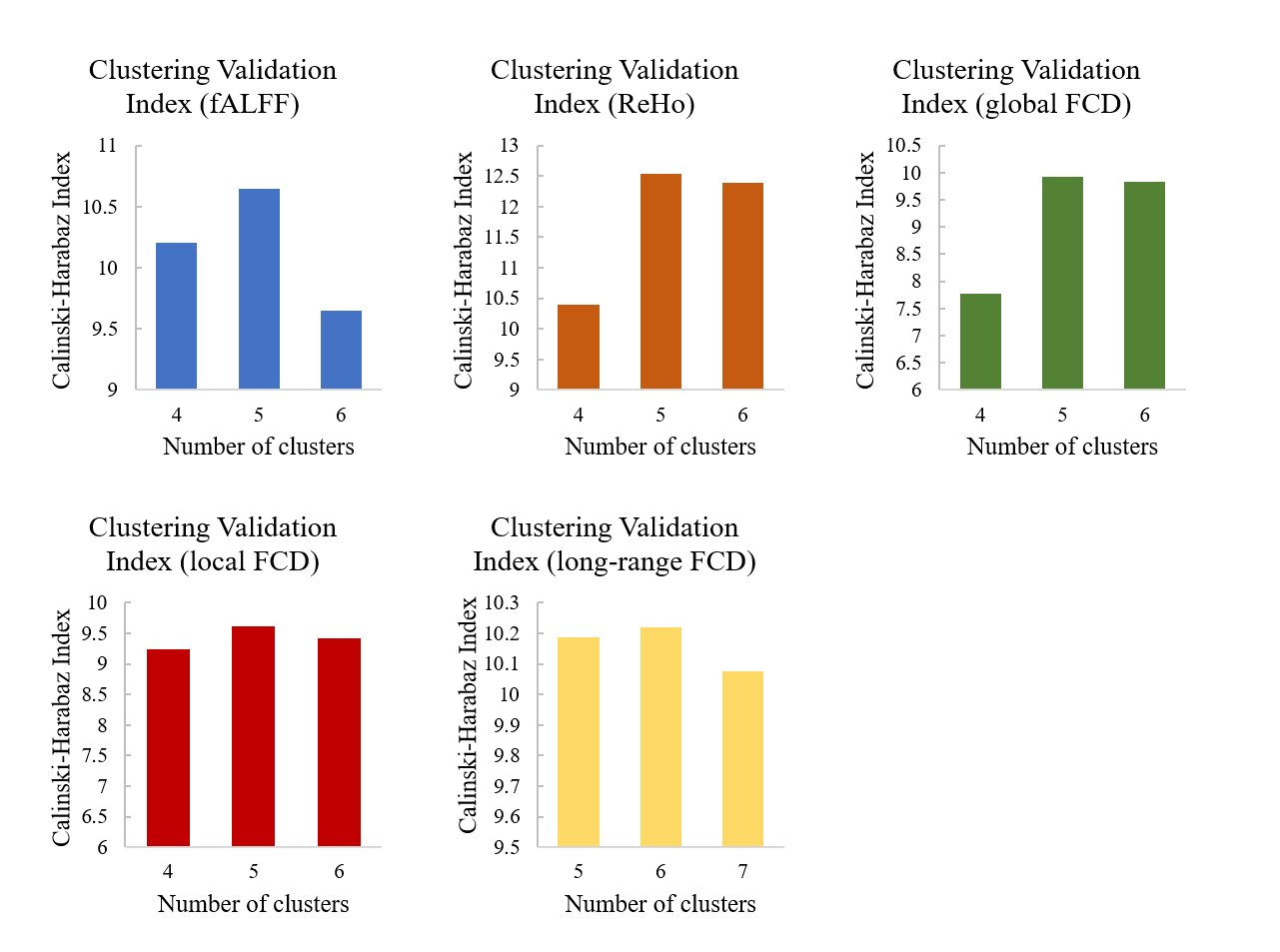


**Fig. S4.** Calinski-Harabaz index in the different number of clusters with APC. With the fixed dumping factor of 0.5 and the preference in the range of 0.9 to 1.1 times the median of similarity, we found that the cluster number of fALFF, ReHo, global FCD, and local FCD ranges from 4 to 6, and the cluster number of the long-range FCD ranges from 5 to 7. In all FIs, the cluster number estimated with the preference as the medium of the similarity matrix got maximum values. Thus, the fALFF, ReHo, global, and local FCD were clustered into 5 states, and the long-range FCD was clustered into 6 states.


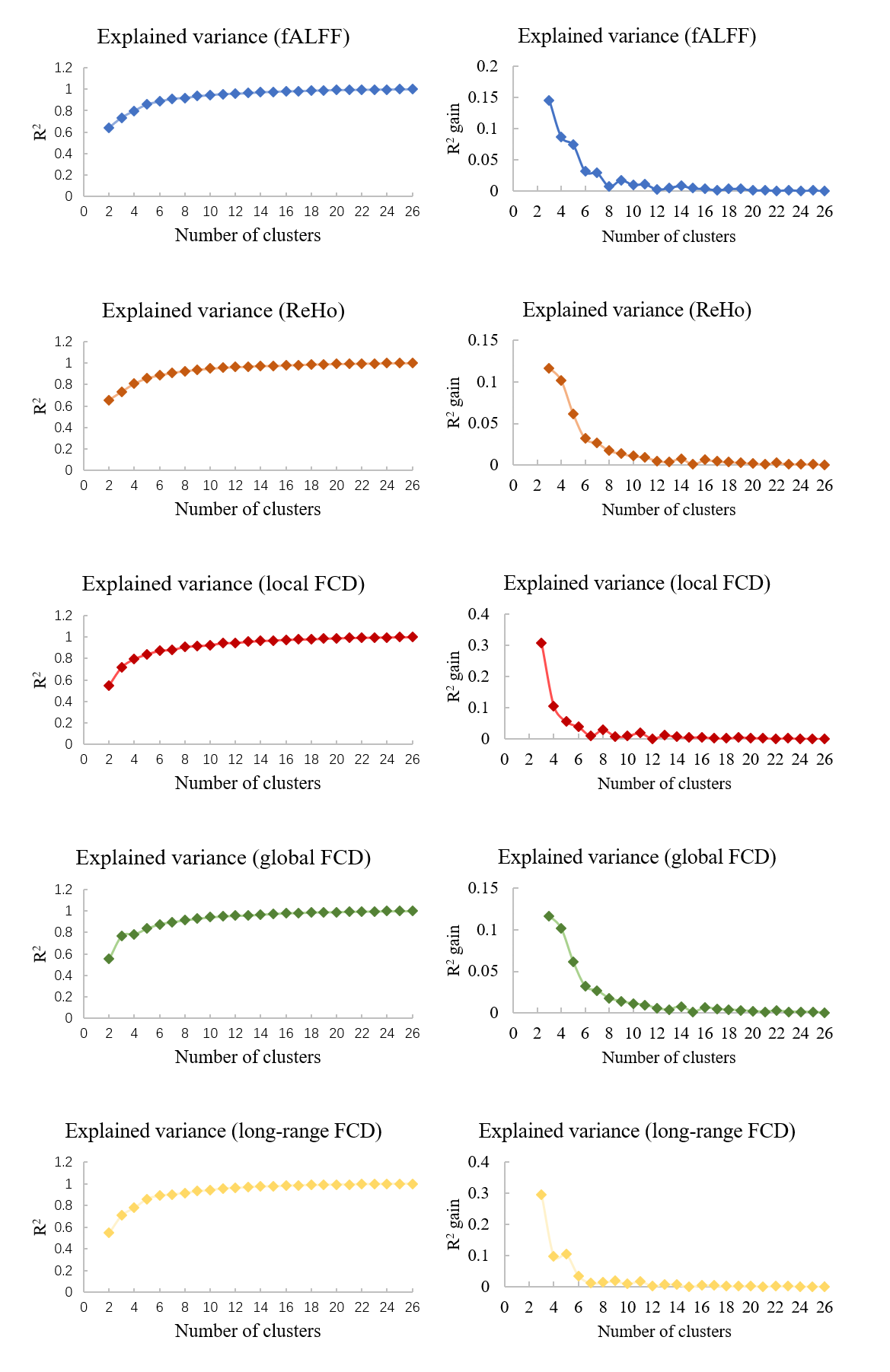


**Fig. S5.** Explained variance and variance gain in the k-means approach. The explained variance curve showed a clear elbow region encompassing the range k =5-7 for fALFF, ReHo, global FCD, and local FCD. The elbow region for long-range FCD encompassed the range k =6-8. Further increasing cluster number after the elbow examined less than 10% of the total variance. Thus, the k-means approach also validated the cluster number of APC.

# Validation with different sliding window lengths

Table S2. Subgroups information with a window length of 6 years.

| subgroup | Duration range(year) | Mean duration | Number |
| --- | --- | --- | --- |
| subgroup_1 | 0-6 | 2.46 | 20 |
| subgroup_2 | 1-7 | 3.63 | 21 |
| subgroup_3 | 2-8 | 4.80 | 20 |
| subgroup_4 | 3-9 | 6.00 | 21 |
| subgroup_5 | 4-10 | 7.33 | 21 |
| subgroup_6 | 5-11 | 8.79 | 19 |
| subgroup_7 | 6-12 | 8.79 | 19 |
| subgroup_8 | 7-13 | 9.35 | 20 |
| subgroup_9 | 8-14 | 10.18 | 17 |
| subgroup_10 | 9-15 | 11.41 | 17 |
| subgroup_11 | 10-16 | 12.88 | 16 |
| subgroup_12 | 11-17 | 14.08 | 13 |
| subgroup_13 | 12-18 | 15.69 | 13 |
| subgroup_14 | 13-19 | 16.47 | 17 |
| subgroup_15 | 14-20 | 17.58 | 19 |
| subgroup_16 | 15-21 | 17.95 | 19 |
| subgroup_17 | 16-22 | 19.20 | 20 |
| subgroup_18 | 17-23 | 20.25 | 20 |
| subgroup_19 | 18-24 | 20.76 | 21 |
| subgroup_20 | 19-25 | 21.60 | 20 |
| subgroup_21 | 20-26 | 22.67 | 18 |
| subgroup_22 | 21-27 | 24.06 | 17 |
| subgroup_23 | 22-28 | 24.47 | 17 |
| subgroup_24 | 23-29 | 25.73 | 15 |
| subgroup_25 | 24-30 | 26.42 | 12 |

Table S3. Subgroups information with a window length of 7 years.

| subgroup | Duration range(year) | Mean duration | Number |
| --- | --- | --- | --- |
| subgroup_1 | 0-7 | 3.22 | 24 |
| subgroup_2 | 1-8 | 4.18 | 24 |
| subgroup_3 | 2-9 | 5.50 | 24 |
| subgroup_4 | 3-10 | 6.64 | 25 |
| subgroup_5 | 4-11 | 7.80 | 24 |
| subgroup_6 | 5-12 | 8.79 | 19 |
| subgroup_7 | 6-13 | 9.19 | 21 |
| subgroup_8 | 7-14 | 9.57 | 21 |
| subgroup_9 | 8-15 | 10.90 | 20 |
| subgroup_10 | 9-16 | 12.10 | 20 |
| subgroup_11 | 10-17 | 13.12 | 17 |
| subgroup_12 | 11-18 | 14.81 | 16 |
| subgroup_13 | 12-19 | 16.47 | 17 |
| subgroup_14 | 13-20 | 17.14 | 21 |
| subgroup_15 | 14-21 | 17.75 | 20 |
| subgroup_16 | 15-22 | 18.65 | 23 |
| subgroup_17 | 16-23 | 19.70 | 23 |
| subgroup_18 | 17-24 | 20.59 | 22 |
| subgroup_19 | 18-25 | 21.13 | 23 |
| subgroup_20 | 19-26 | 22.00 | 22 |
| subgroup_21 | 20-27 | 23.29 | 21 |
| subgroup_22 | 21-28 | 24.28 | 18 |
| subgroup_23 | 22-29 | 24.95 | 19 |
| subgroup_24 | 23-30 | 25.73 | 15 |


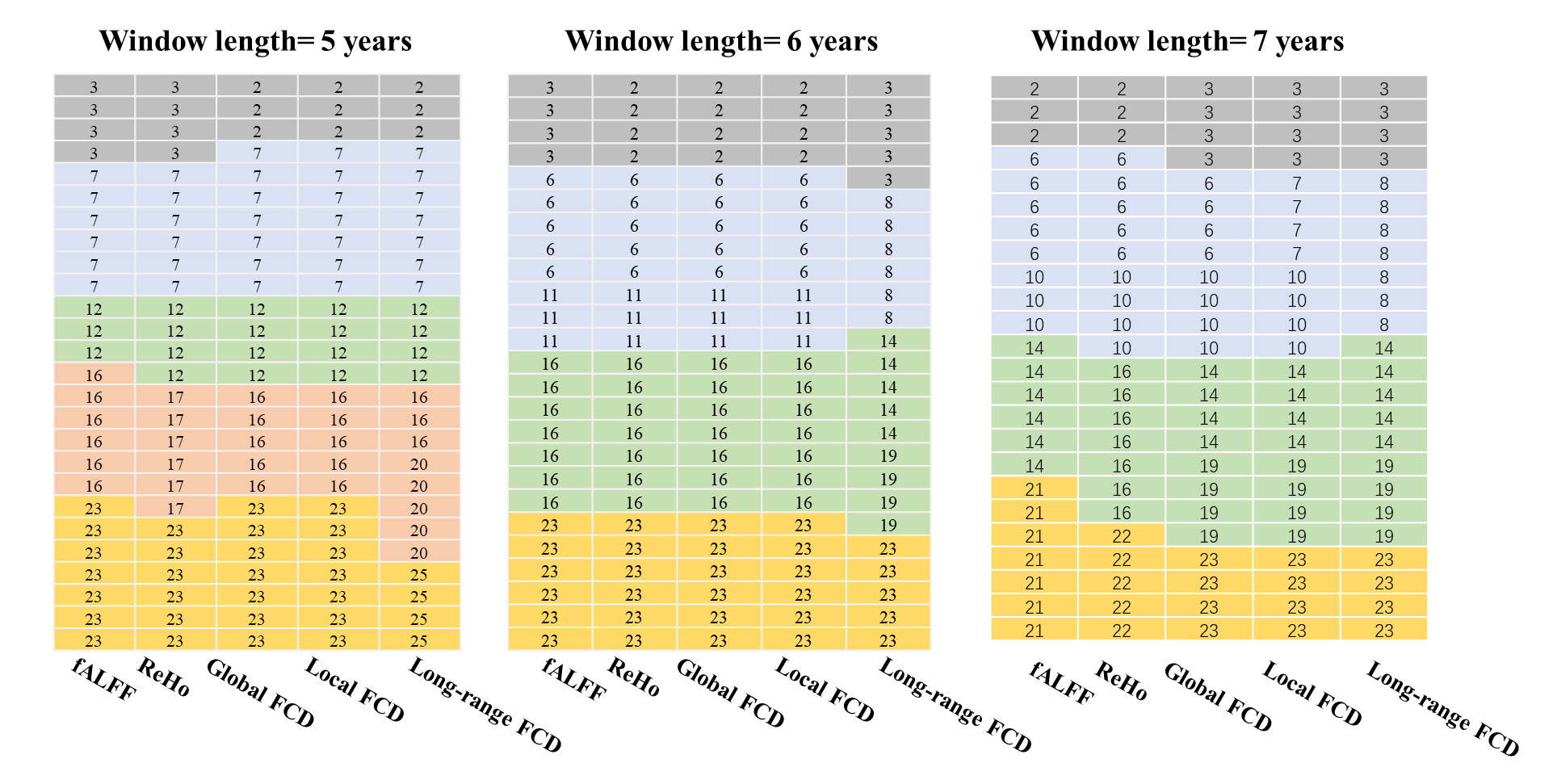


**Fig. S6.** APC results and alignment with different window lengths. After the alignment based on the temporal overlap and spatial similarity, we finally acquired progressive stages. For a convenient view, every stage was marked with one specific color. Four stages were obtained with window lengths of 6 and 7 years.


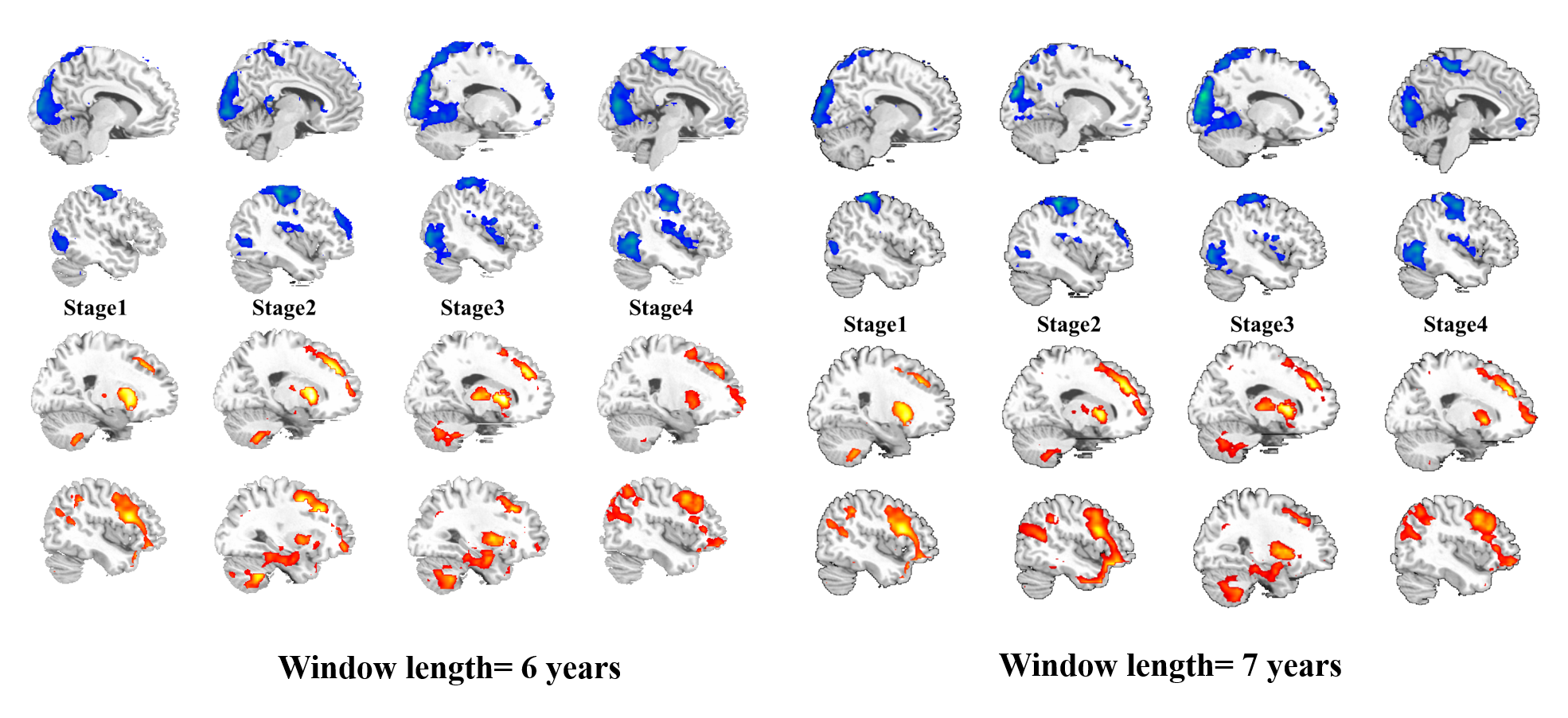


**Fig. S7.** Stage patterns in with window lengths of = 6 years and 7 years. Four stages were obtained with window lengths of 6 and 7 years, consistently suggesting a hypo-functionality trajectory from lower-order cortices to insula to higher-order cortices in the default mode network and a hyper-functionality trajectory from the subcortical to the hippocampus to higher-order cortices in the frontoparietal network.

# Association between progressive dysfunction and functional gradients

The cerebral function gradient maps were obtained from our previously published work (Fig. S8. A, B), which was calculated using the method of Margulies et al. We employed both functional gradient 1 and gradient 2 in this analysis. First, we did not find a linearly increased correlation between dysfunction and gradient 1 or gradient 2. Correlating with function gradient 1, we found that the dysfunction pattern showed an increased positive correlation from stage 1 to stage 2, then decreased gradually from stage 2 to stage 4, and finally raised again from stage 4 to stage 5 (Fig. S8. C, E). Correlating with function gradient 2, the dysfunction pattern showed an increased negative correlation from stage 1 to stage 2, then decreased gradually from stage 2 to stage 3, and finally raised again from stage 3 to stage 5 (Fig. S8. D, F). The present findings suggested the higher-order function impairment of schizophrenia is aggravated in the later stage of the disease progression, but the evolution process of nonlinear changes. Interestingly, the relative ratio of correlation with gradient 1 (demeaned) and correlation with gradient 2 (demeaned) showed the same trend as the PANSS positive scores across stages (Fig. S8. G), which might imply an association between individual behaviors and the integrative brain functional gradient.


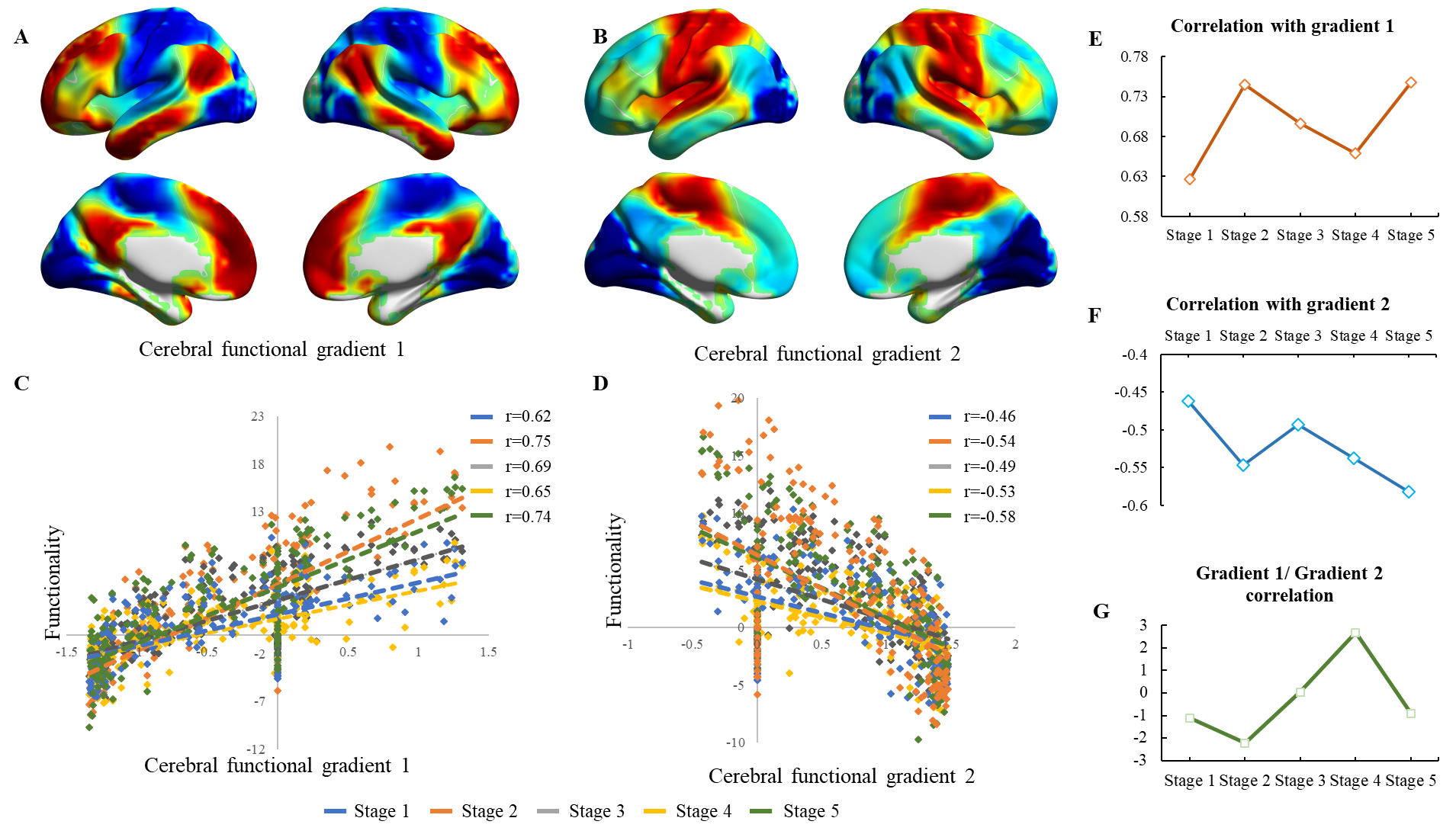


**Fig. S8.** Correlation between progressive dysfunction and cerebral function gradients. (A, B) Cerebral functional gradient map from our previously published work. (C, D, E, F) Spatial correlation between progressive dysfunction maps and cerebral functional gradients. (G) We first demeaned the correlation between progressive dysfunction maps and cerebral functional gradient 1 and gradient 2 and then calculated the ratio of correlation.

# Neuroimage PLS1 loadings associated with symptoms


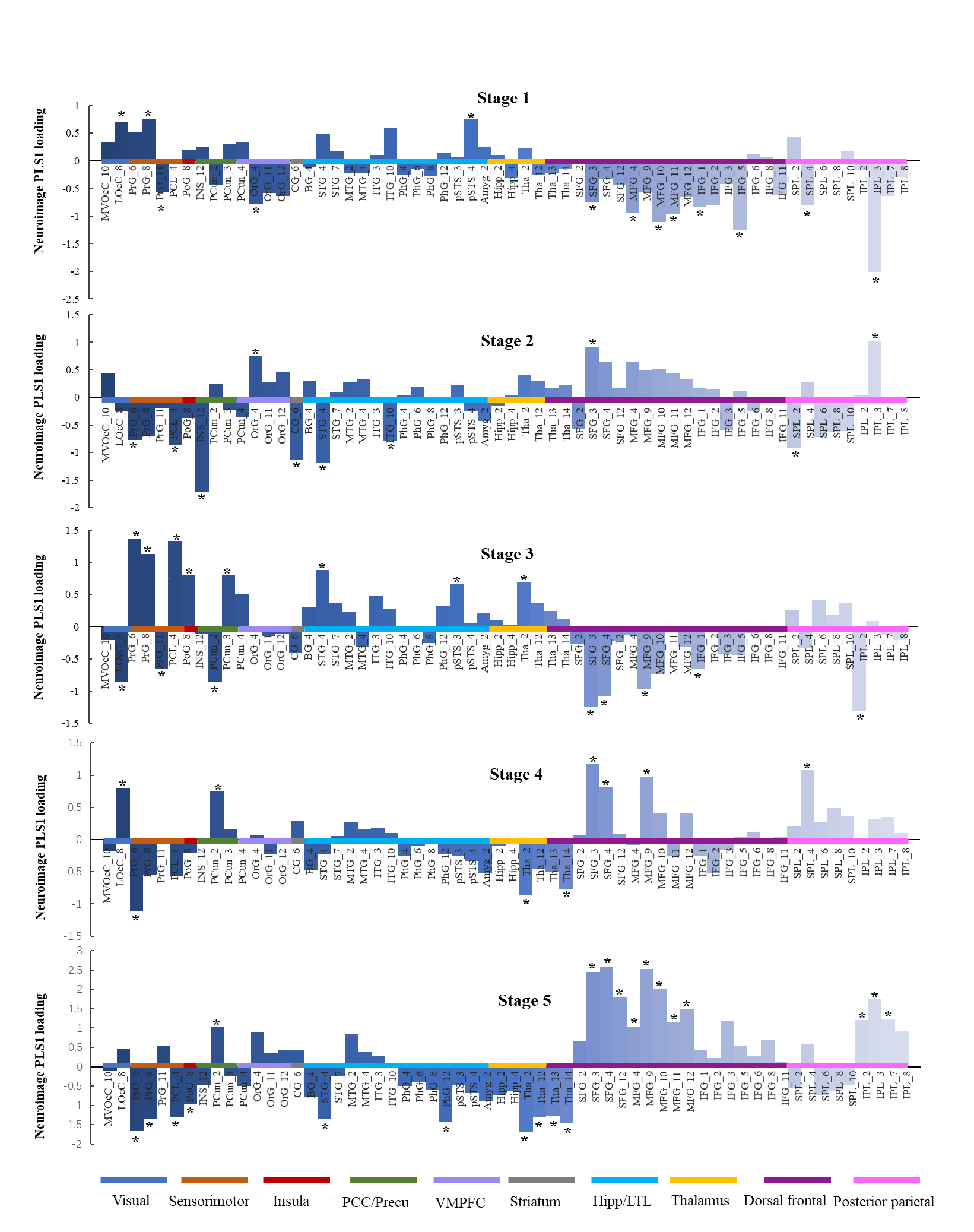


**Fig. S9.** Neuroimaginge PLS1 loadings across stages. We extracted the PLS1 loadings of all sixty subregions located in the hyper-functionality and hypo-functionality trajectories. The asterisk (*) represents p<0.05 FDR corrected using a permutation test.

# Control analyses

## Drug effects


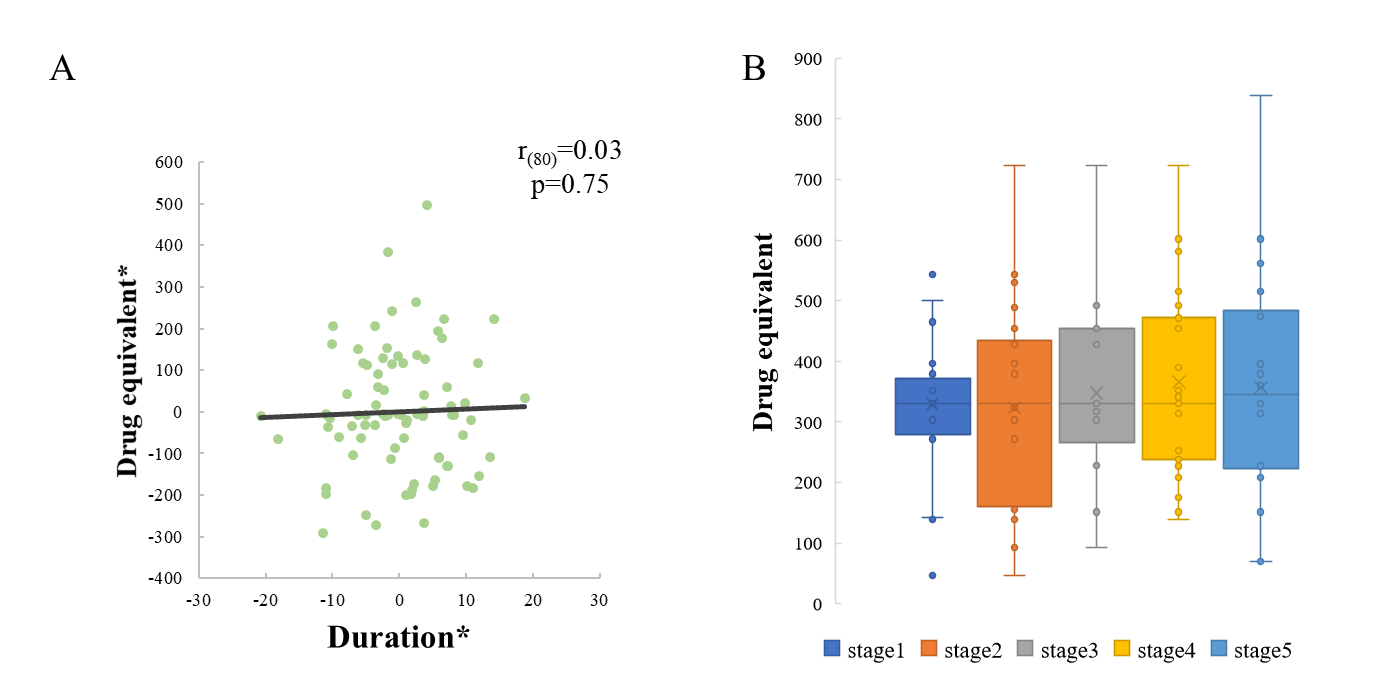


**Fig. S10.** Relationship between illness disease duration and drug equivalent. (A) No significant (p>0.05) correlation was found between illness duration and drug equivalent across all patients in this study. The asterisk (*) indicates residual after-regression nuisance covariates (age, gender, mFD, and TIV). (B) No significant (p>0.05) difference was found among patients in different stages.


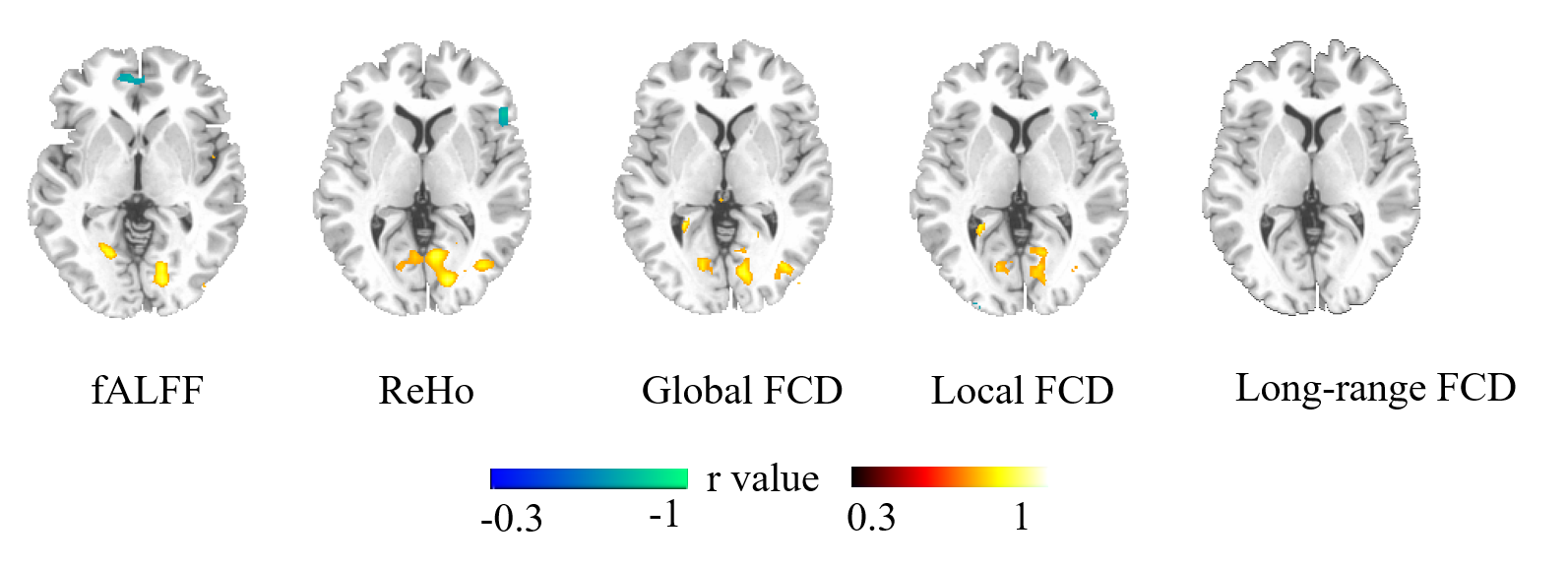


**Fig. S11.** Correlation between FIs and drug equivalent (|r| > 0.3). Except for the long-range FCD, the other four indicators positively correlated to illness duration in a cluster located in visual cortices.


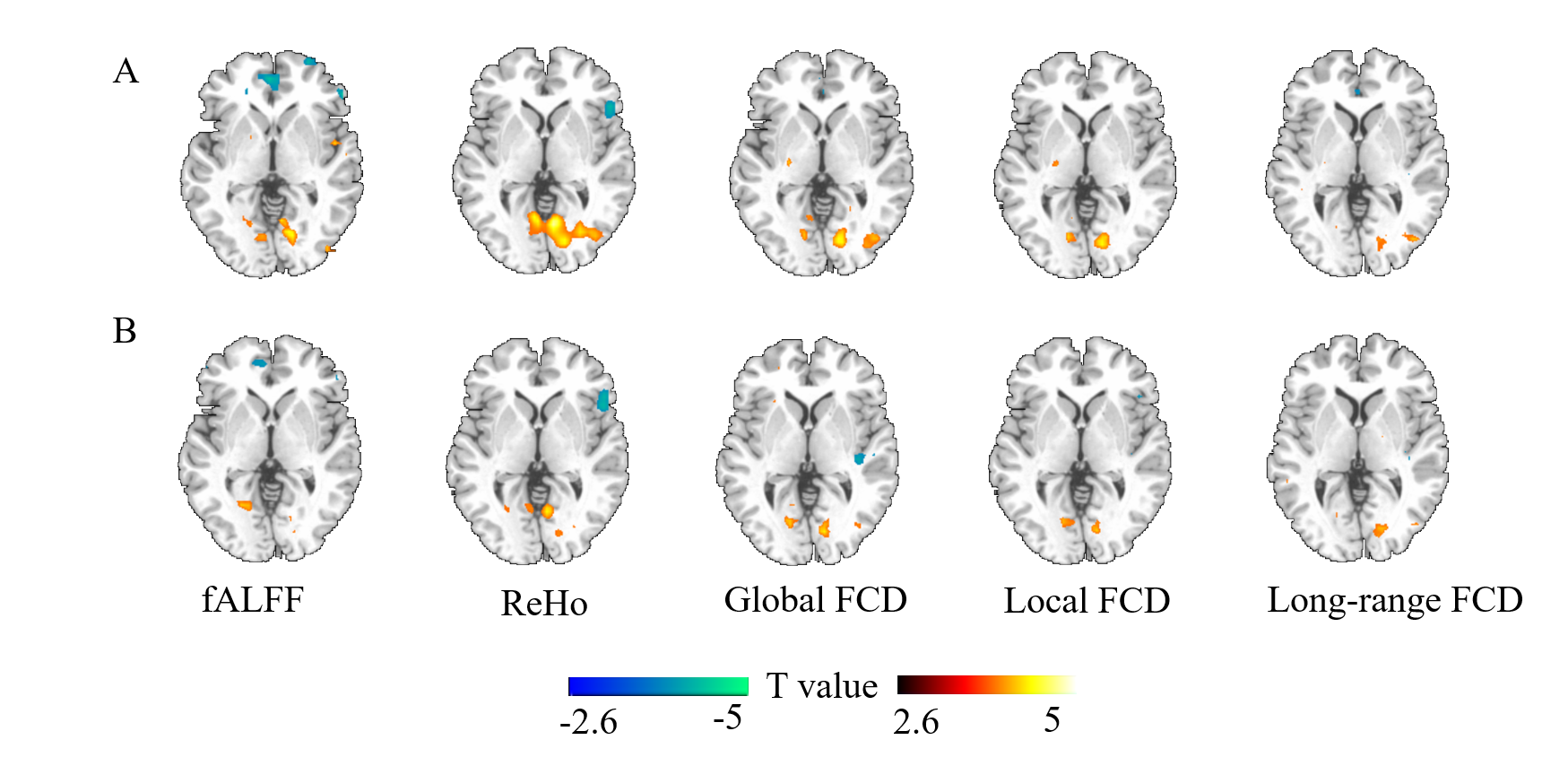


**Fig. S12.** Comparison of FIs between patients with high and low drug equivalents. (A) Patients with the top 20% drug equivalent showed increased FIs in visual cortices relative to panties with a down 20% drug equivalent (p<0.001, uncorrected). (B) Patients with the top 30% drug equivalent showed increased FIs in visual cortices relative to panties with a down 30% drug equivalent (p<0.001, uncorrected).

## Structure effects


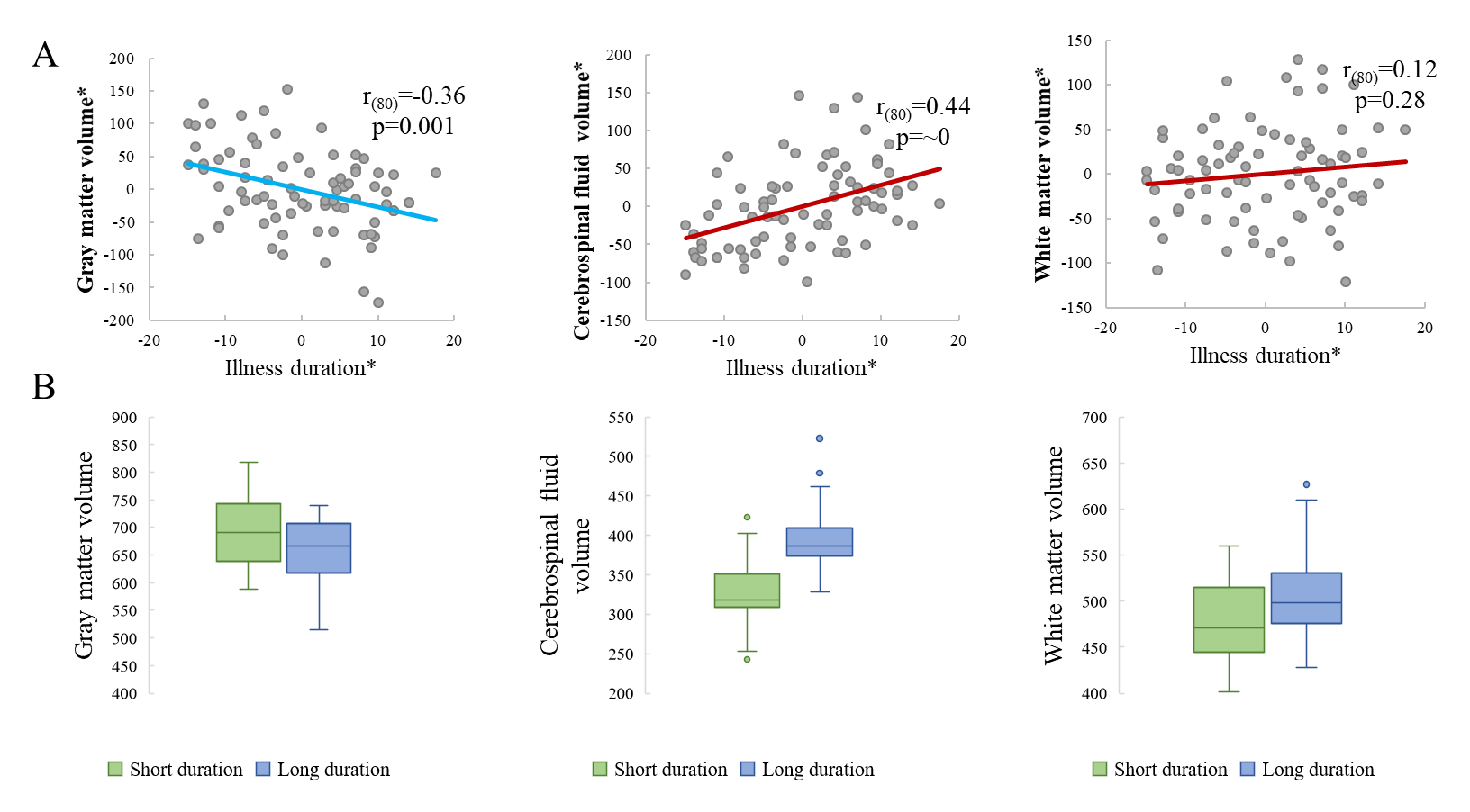


**Fig. S13.** Relationship between illness disease duration and brain structural features. (A) Correlation (Pearson’s coefficient) between illness duration and brain structural features. As shown, a significant negative correlation (r=-0.36, p=0.001) was observed between grey matter volume and illness duration. The cerebrospinal fluid volume is significantly positively correlated to illness duration (r=0.44, p=~0). The white matter volume did not show a significant correlation with duration (r=0.12, p=0.28). The asterisk (*) indicates residual after-regression nuisance covariates (age, gender, mFD, and TIV). (B) Comparisons of brain structural features between patients with short duration and long duration. Subgroup comparisons also suggested a significant decrease in grey matter volume and an increase in cerebrospinal fluid volume in patients with long duration relative to short duration.

# Association with dopamine function

All case-control t-maps of FIs showed a significantly positive correlation (p_spin_<0.05) with dopamine synthesis (Fig. S14). Case-control t-maps of progressive stages also showed a significant correlation with dopamine features (Fig. S15).


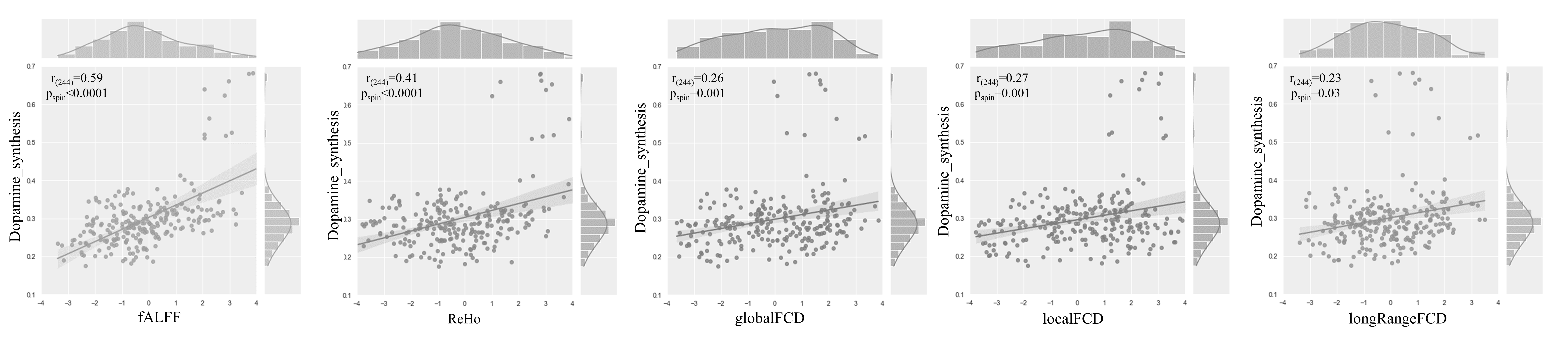


**Fig. S14.** Spatial correlation between case–-control t-maps of five functional indicators and dopamine synthesis (*p_spin_< 0.05)*.


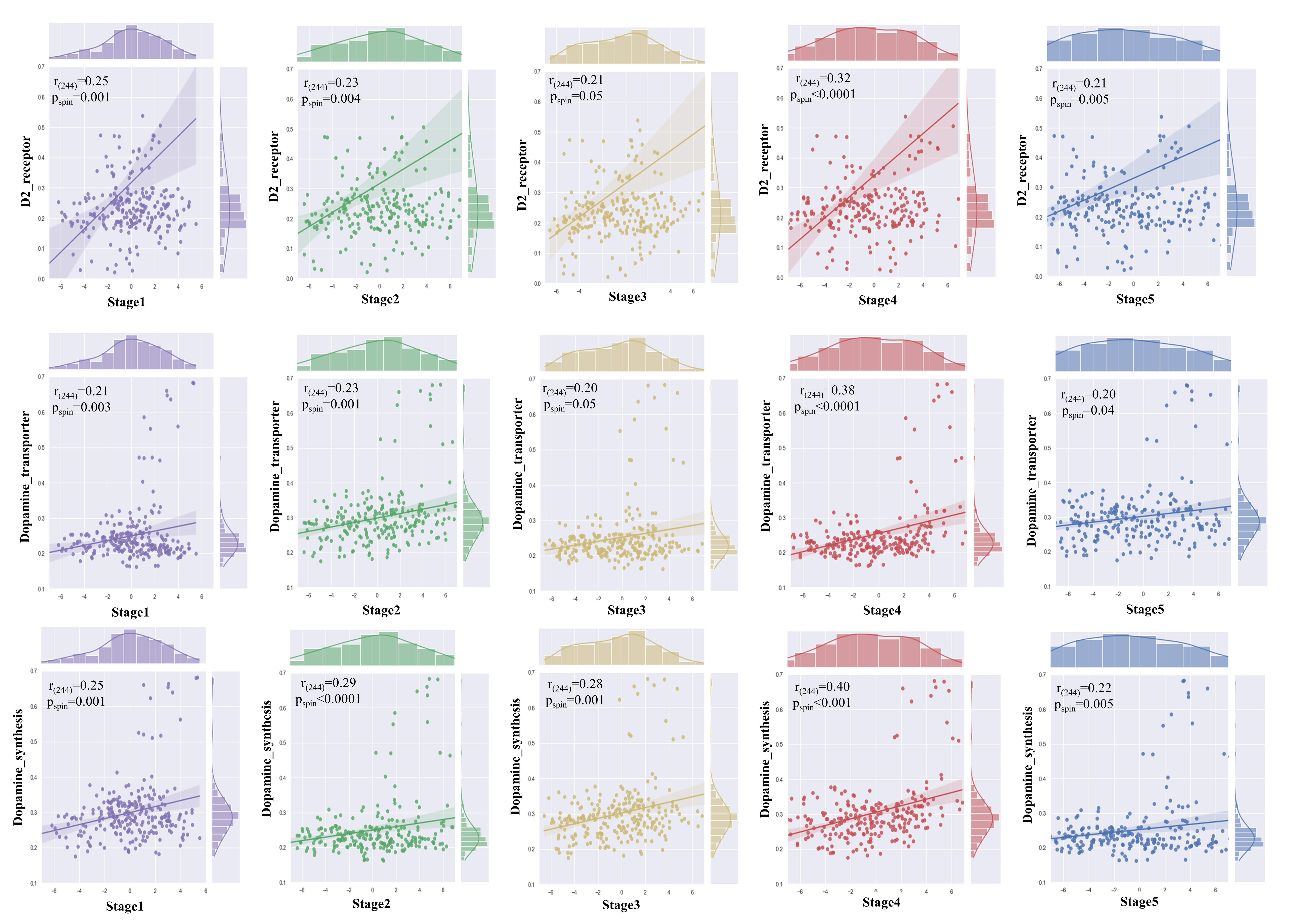


**Fig. S15.** Spatial correlation between case–-control z-maps of five stages and dopamine synthesis (*p_spin_* < 0.05)

# Correlation between progressive stages and PLS1

Fig. S16 showed a significant correlation between the z-maps of progressive stages and their PLS1 maps.


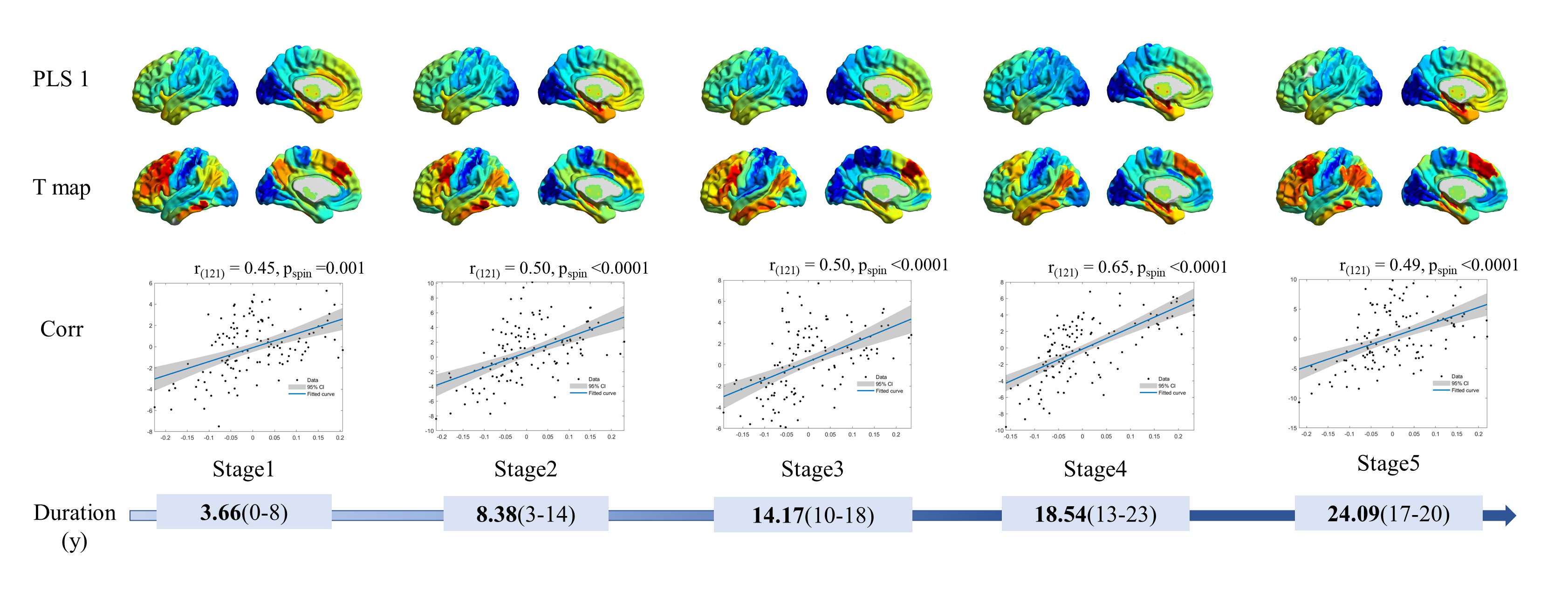


**Fig. S16.** Correlation between PLS1 maps and case–-control t-maps in all progressive stages of the disease (labeled with different disease durations). As shown, significant positive correlations were found in all stages, suggesting a fine potential genetic representation of functionality phenotypes.

# Enrichment with merged PLS1 genes of all stages

Enrichment analyses were also conducted with merged PLS1+ and PLS1- genes of all stages. Enriched terms and enrichment networks across stages were shown below (Fig. S17-18). The results of PLS1- were illustrated in the main context.

**
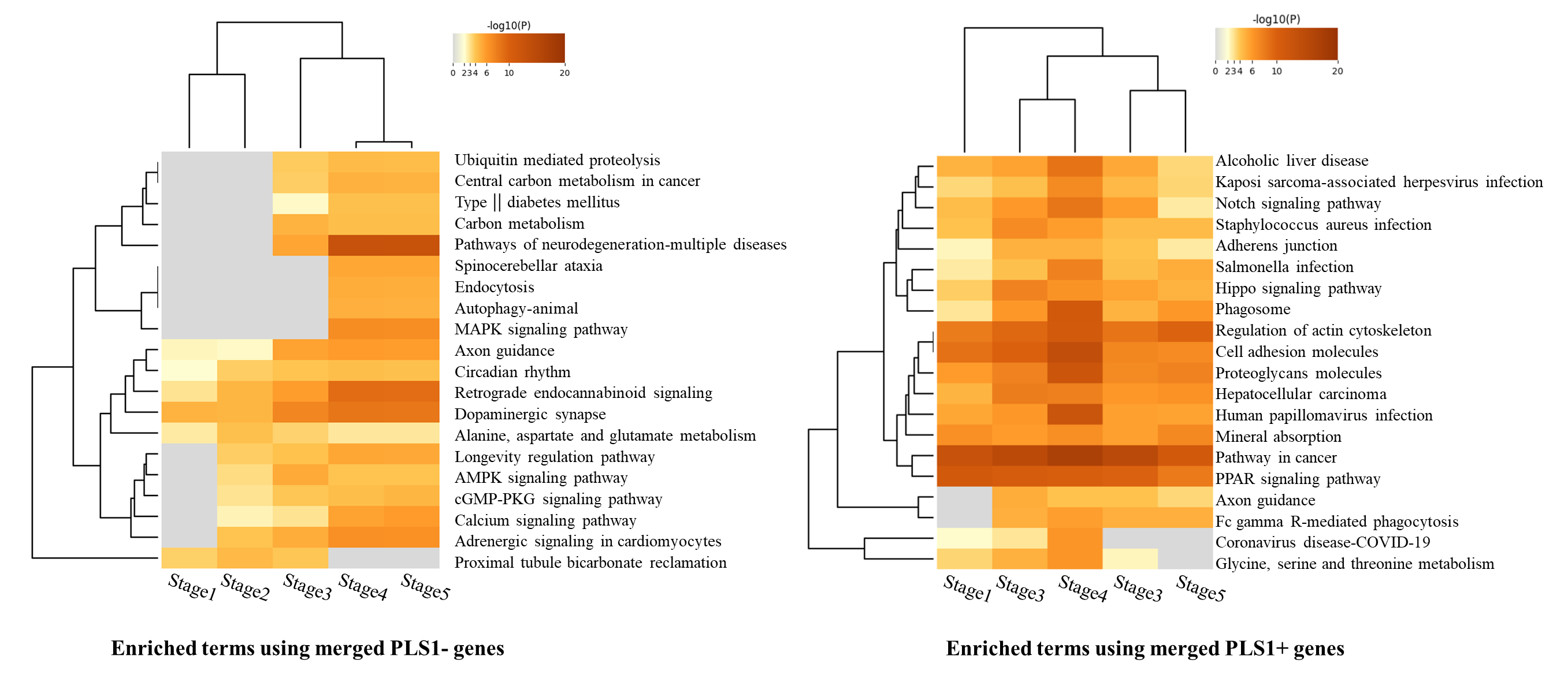
**

**Fig. S17.** Enriched terms across stages and PLS1 (Z < −-5) gene lists, colored by p-values.


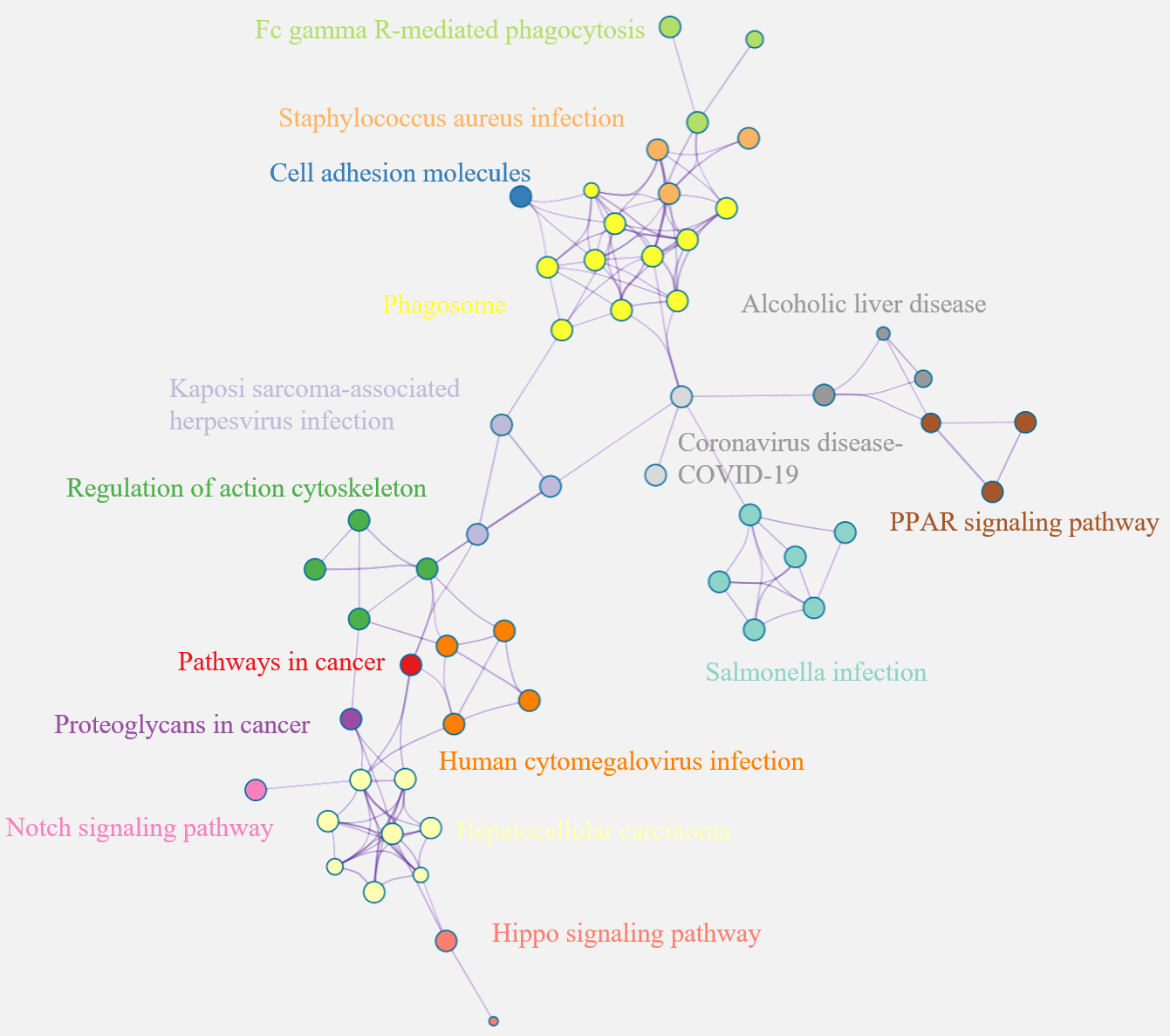


**Fig. S18.** KEGG enrichmented network from merged PLS1+ (Z > 5) genes of the five progressive stages. The top three terms with the best p values include “Pathways in cancer” (-long10(p)=18.50), “Cell adhesion molecules” (-long10(p)=13.74), and “Regulation of actin cytoskeleton” (-long10(p)=12.56).
