# Supplementary material for: Progressive trajectories of schizophrenia across symptoms, genes, and the brain
Source: BMC Med. 2023 Jul 3;21:237. doi: 10.1186/s12916-023-02935-2 (PMC10318676; doi:10.1186/s12916-023-02935-2)
Supplement: Supplementary file 3 — Additional file 3: Fig. S19. Case–control t-maps of functional indicators and their spatial associations with PLS1. Fig. S20. Genes related to the case–control t-maps of the five functional indicators. Fig. S21. KEGG networks using merged PLS1 genes. Fig. S22. Case–control t-maps and PLS1 maps of FIs in the discovery and replication datasets. Fig. S23. KEGG networks using merged PLS1 genes. [file 12916_2023_2935_MOESM3_ESM.docx]

***Supplementary materials for “Progressive trajectories of schizophrenia across symptoms, genes, and the brain*”**

# Results

# Non-progressive analysis in the discovery cohort

## Case-control differences in FIs

All case-control t-maps positively correlated with the PLS1 (Fig. S19). All FIs showed a common decrease in the sensorimotor and visual cortices and a common increase in the striatum (including putamen, and pallidum). Sensorimotor cortices include precentral and postcentral gyrus (PPG) and supplementary motor regions (SMA). Functional connectivity characteristics showed a common increase in the dorsal medial prefrontal cortex (DMPFC) and dorsal lateral prefrontal cortices (DLPFC). Decreased FCD values (global, local, and long-range) were observed in ventral medial prefrontal cortices (VMPFC) and increased FCD values (global and long-range) were observed in the cerebellum and hippocampus. Increased local FCD was found in a cluster located in the lateral occcipito-temporal junction (LOTJ).


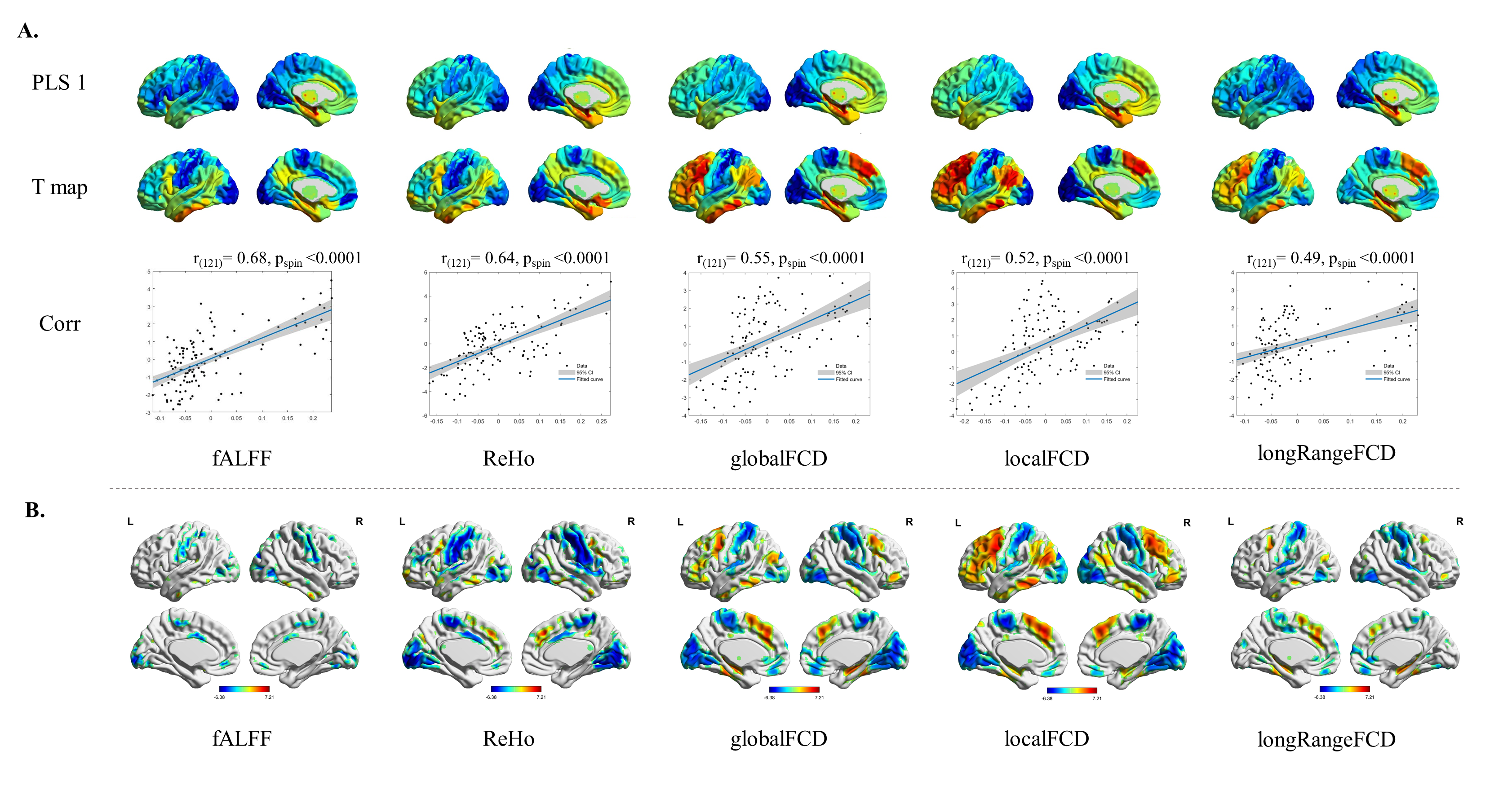


**Fig. S19.** Case–-control t-maps of functional indicators and their spatial associations with PLS1. (A) A significantly positive correlation was observed between un-threshold case-control t-maps of functional indicators and PLS1. (B) Significant case-control differences were detected with a threshold of p<0.05 with FDR correction.

## Genes related to case-control differences

Five case-control t-maps positively correlated with their PLS1 (mean r=0.58, ranging from 0.49-0.68). The PLS1 of fALFF contains 36 of 52 schizophrenia-related genes from in situ hybridization, including 28 genes positively and 8 genes negatively correlating with the case-control t-map (Fig. S20). The PLS1 of ReHo contains 29 of 52 schizophrenia-related genes from in situ hybridization, including 18 genes positively and 11 genes negatively correlating with the case-control t-map. Global, local, and long-range FCD contains 31, 23, and 31 of 52 schizophrenia-related genes respectively, including 30, 16, and 25 genes negatively correlating and 1, 7, and 6 genes positively correlating to case-control t-maps. The gene expression with the most correlation (positive and negative) to five case-control t-maps was a plot in Fig. S20 respectively. Notably, the gene expression of KCNN3 (4/5) and HTR2C (1/5) showed the most positive correlation with case-control t-maps. The gene expression most negatively correlated with case-control t-maps included AH1(2/5), PCNT (1/5), PVALB (1/5), and SPTBN4(1/5).


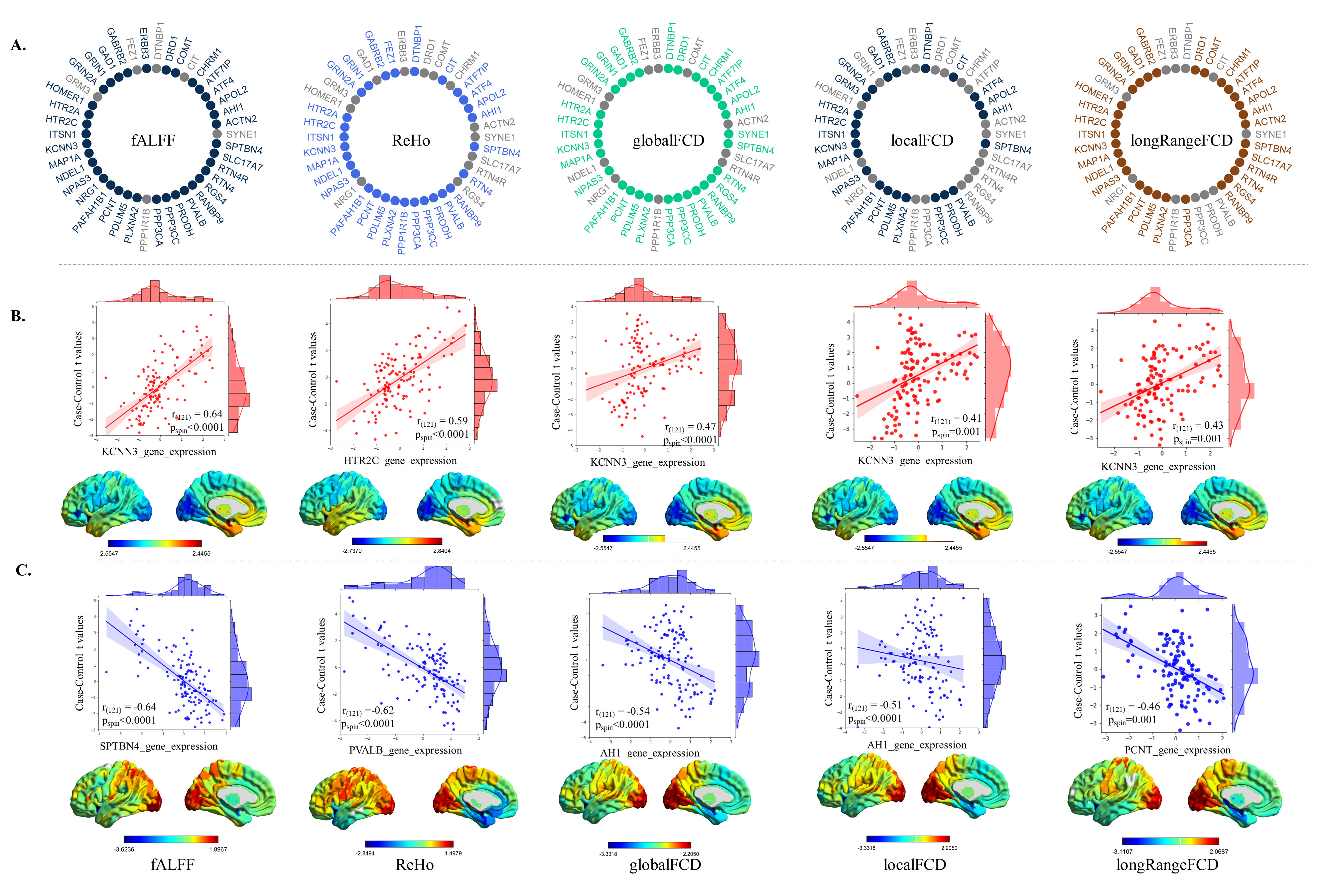


**Fig. S20.** Genes related to the case–-control t-maps of the five functional indicators. (A) Overlap between PLS1 genes and schizophrenia-related genes from in situ hybridization. (B) Most positive correlation between case-control t-maps and certain gene expression. (C) Most negative correlations between case-control t-maps and certain gene expressions.

## Enrichment network using PLS1 genes in the discovery cohort

The duration-sliding progressive analysis is an innovative approach, even though we suggested it is powerful to uncover the trajectory of the disease. The non-progressive analysis provided overall results despite the duration progression, showing mixed effects in schizophrenia. It is necessary to perform additional analysis in a non-progressive way, which could verify the enrichment results of the progressive analysis. Progressive findings in specific stages might be further highlighted when compared with findings from the non-progressive analysis. Besides, both the PLS1+/PLS1- genes were input to enrichment analysis, trying to provide more specific information to understating gene involvement in the disease progression (Fig. S21).


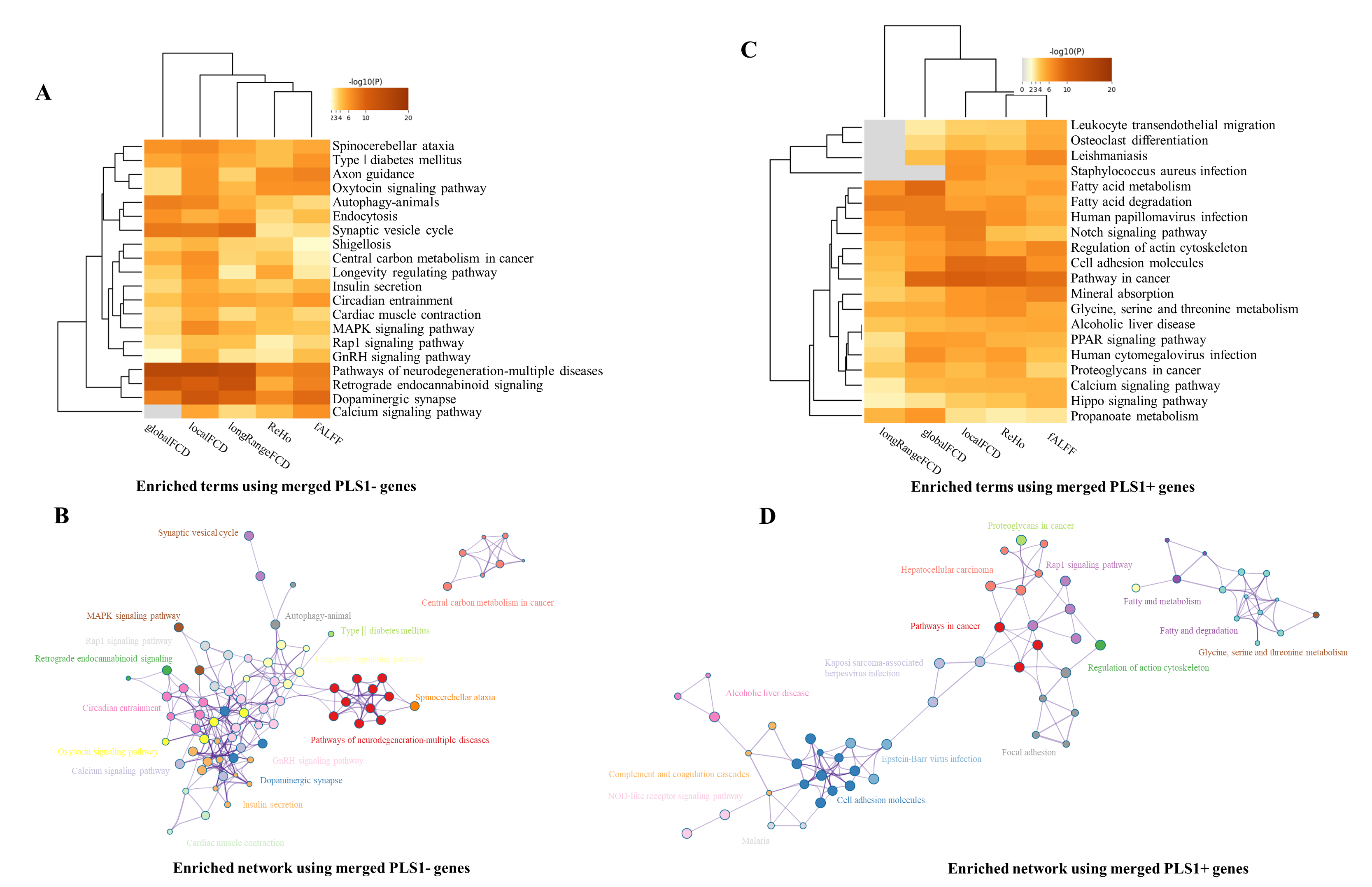


**Fig. S21.** KEGG networks using merged PLS1 genes. (A) Enriched terms across PLS1- (Z<-5) gene lists, colored by p-values. As shown, Pathways of neurodegeneration - multiple diseases” and “Dopaminergic synapse” terms have the highest significance. (B) The enrichment network for merged gene lists of PLS1- genes (Z <-5) of FIs. The top three terms with the best p values include “Dopaminergic synapse”, “Alzheimer's disease”, and “Pathways of neurodegeneration - multiple diseases”. (C) Enriched terms across FIs PLS1- (Z>5) gene lists, colored by p-values. As shown, “Pathways in cancer” and “Cell adhesion metabolism” terms have the highest significance. (D) The enrichment network for merged gene lists of PLS1+ (Z>5) genes of FIs. The top three terms with the best p values include “Dopaminergic synapse”, “Alzheimer's disease”, and “Pathways of neurodegeneration - multiple diseases”.

# Validation analysis in a replication cohort

The findings of the replication cohort showed highly consistent with the findings of the discovery cohort, which further verified the reliability of the present study (Fig. S22-23).

A shared enrichment network in both cohorts uncovered the involvement of dopaminergic synapse, neurodegeneration, neurodevelopment, immune, and cancer genetic factors in schizophrenia. Specifically, we found that the PLS1+ genes seem to be linked with cancer, immune, infection, and metabolism factors, and the PLS1 genes seem to be linked with synaptic systems (dopaminergic-centered), neurodevelopment, and neurodegeneration factors.

## Case-control differences of FIs


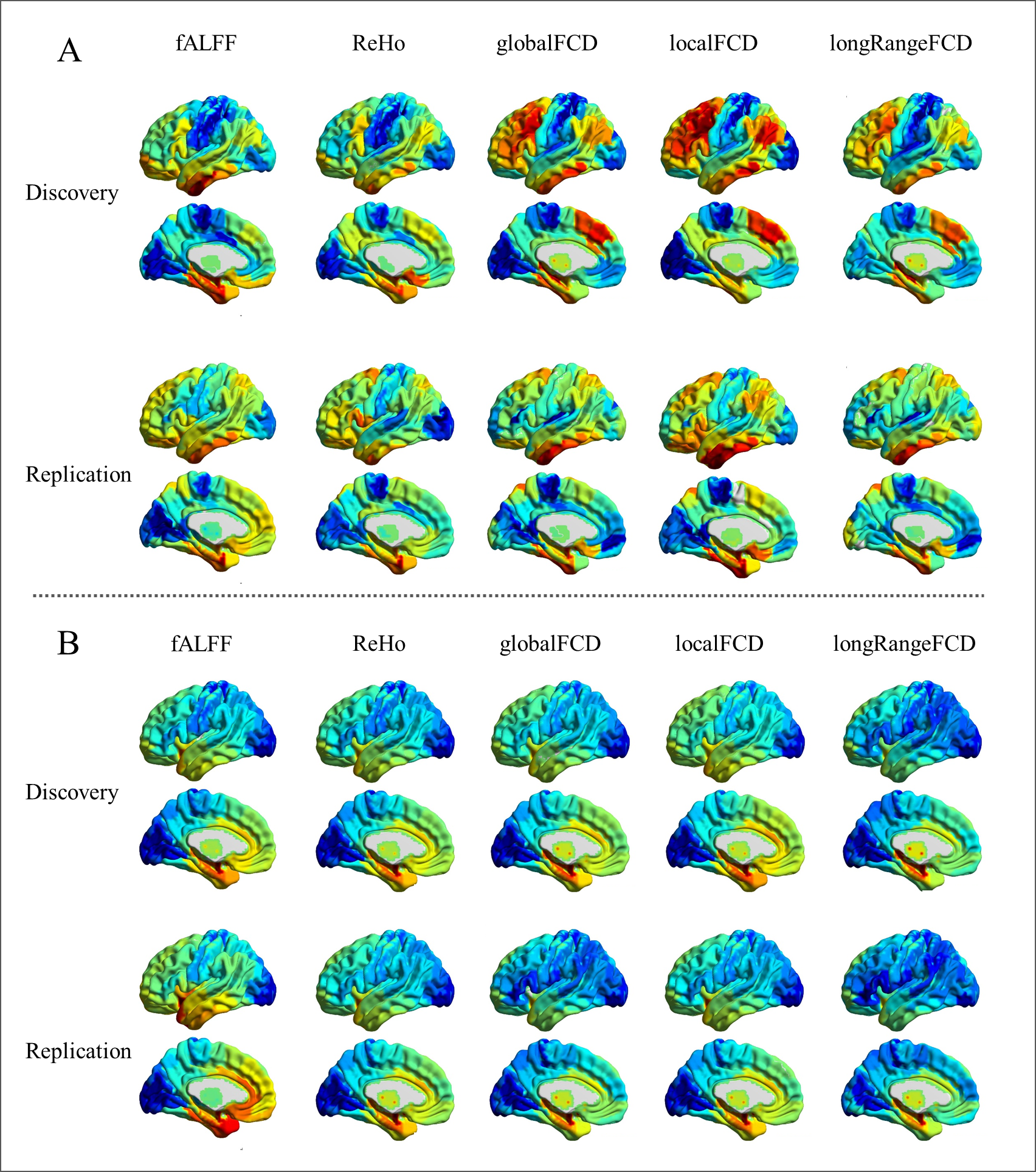


**Fig. S22.** Case-–control t-maps and PLS1 maps of FIs in the discovery and replication data sets. (A) High consistency was found across datasets in case-control t-maps of FIs. (B) High consistency of PLS1 maps of FIs was found across datasets, which validated the results revealed in the discovery cohort.

## Enrichment network using PLS1 genes


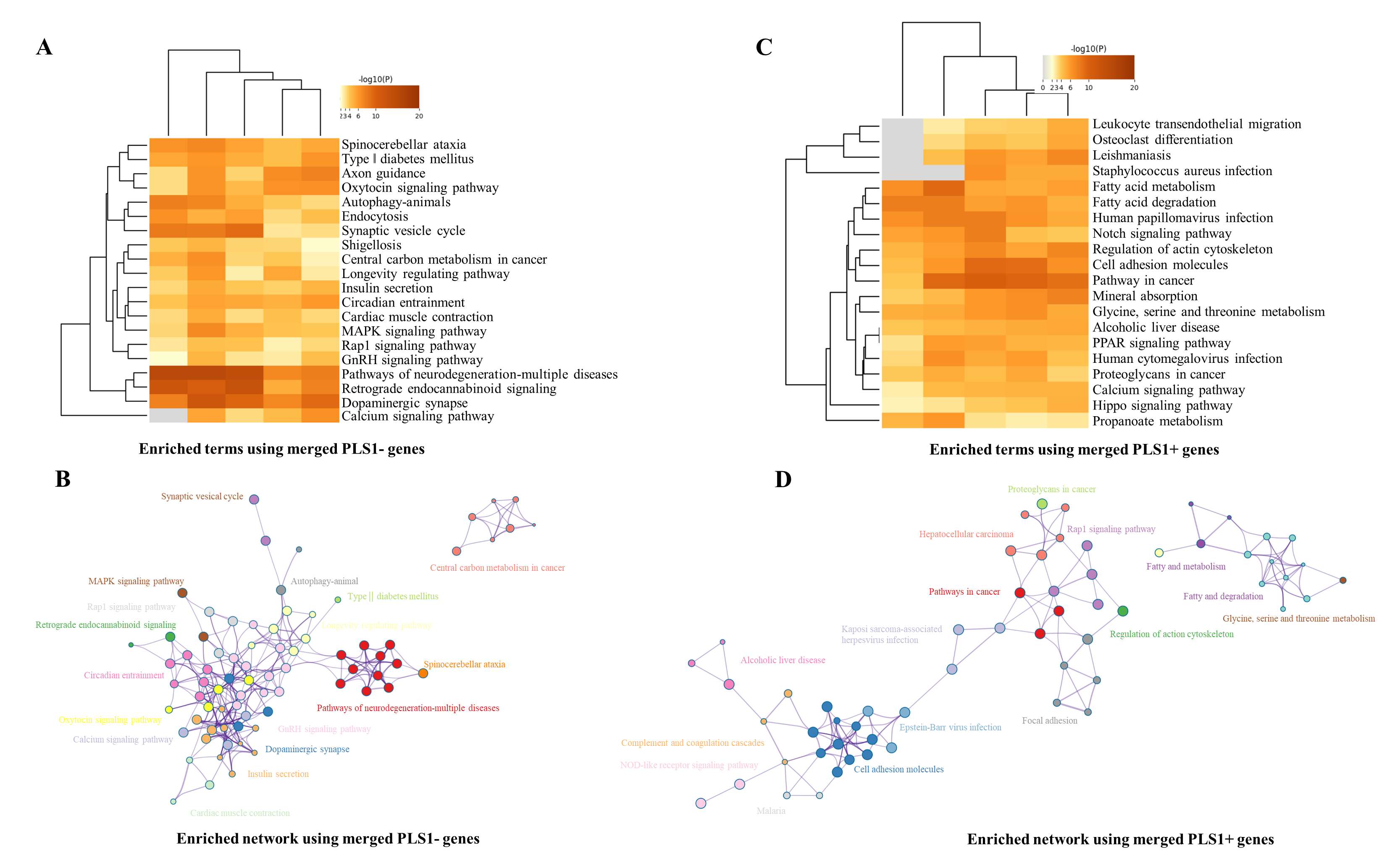


**Fig. S23.** KEGG networks using merged PLS1 genes. (A) Enriched terms across PLS1- (Z<-5) gene lists of FIs, colored by p-values. (B) The enrichment network from merged PLS1- (Z<-5) genes of FIs in the replication dataset. The top three terms with the best p values include “Pathways of neurodegeneration - multiple diseases” (-log10(p)=19.56), “Dopaminergic synapse” (-log10(p)=14.1), and “Retrograde endocannabinoid signaling” (-log10(p)=13.09). (C) Enriched terms across PLS1+ (Z>5) gene lists of FIs, colored by p-values. (D) The enrichment network from merged PLS1+ (Z>5) genes of FIs in the replication dataset. The top three terms with the best p values include “Pathways in cancer” (-log10(p)=10.61), “Fatty acid metabolism” (-log10(p)=9.29), and “Cell adhesion molecules” (-log10(p)=9.19).
